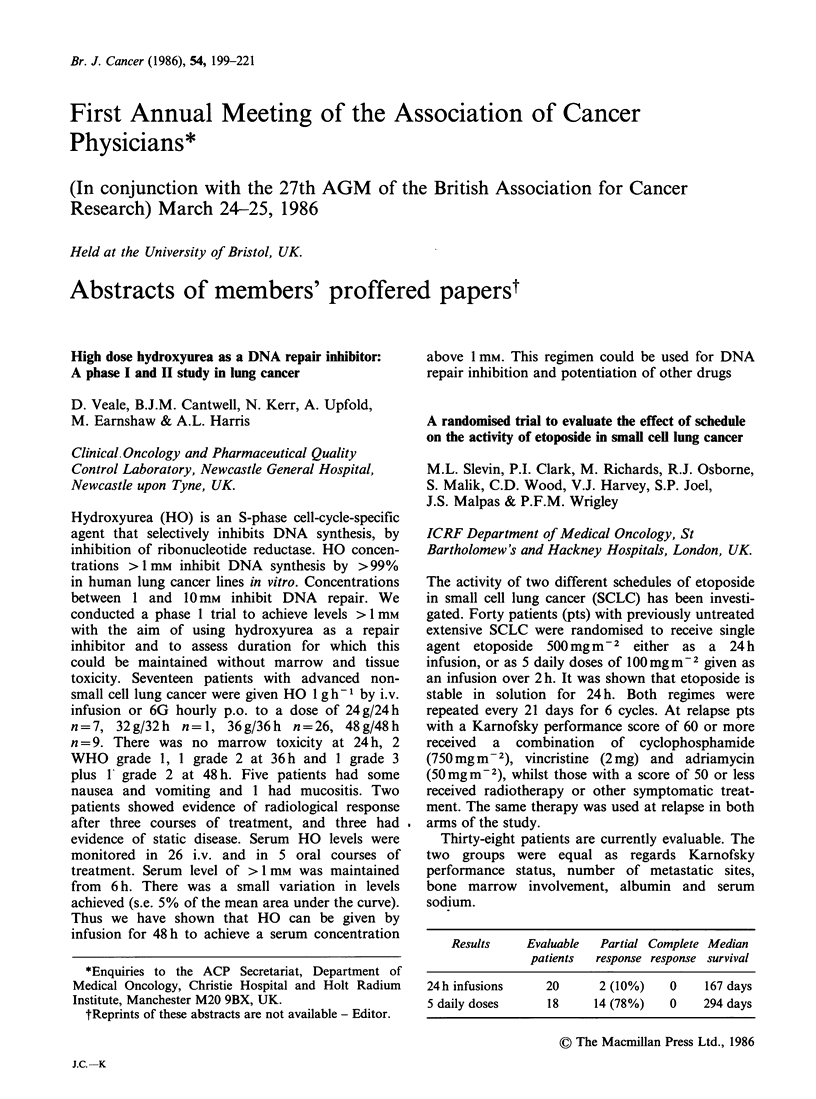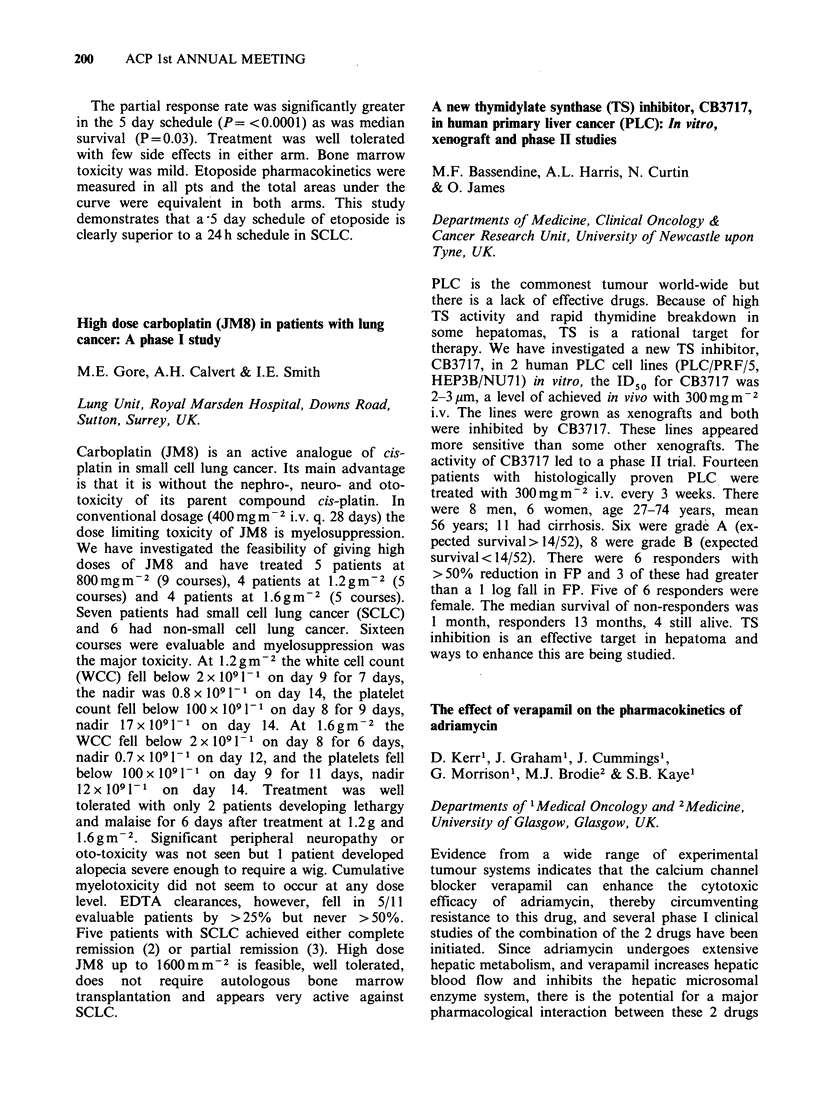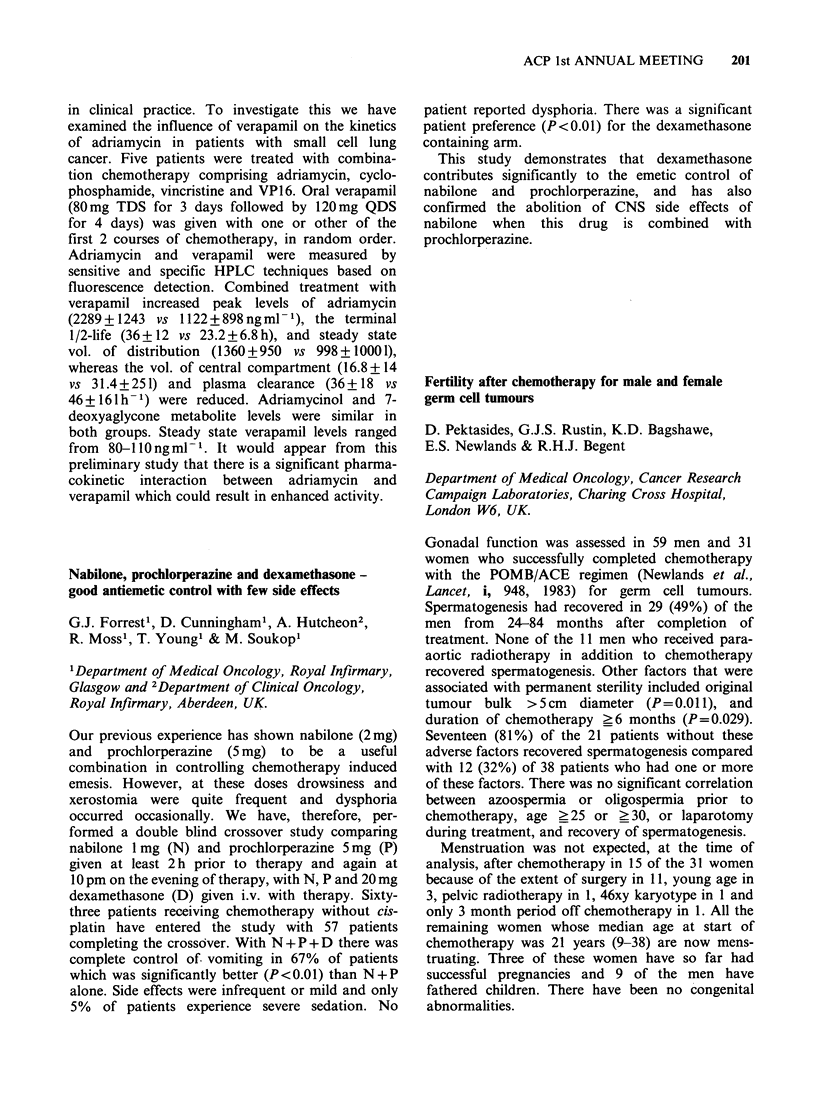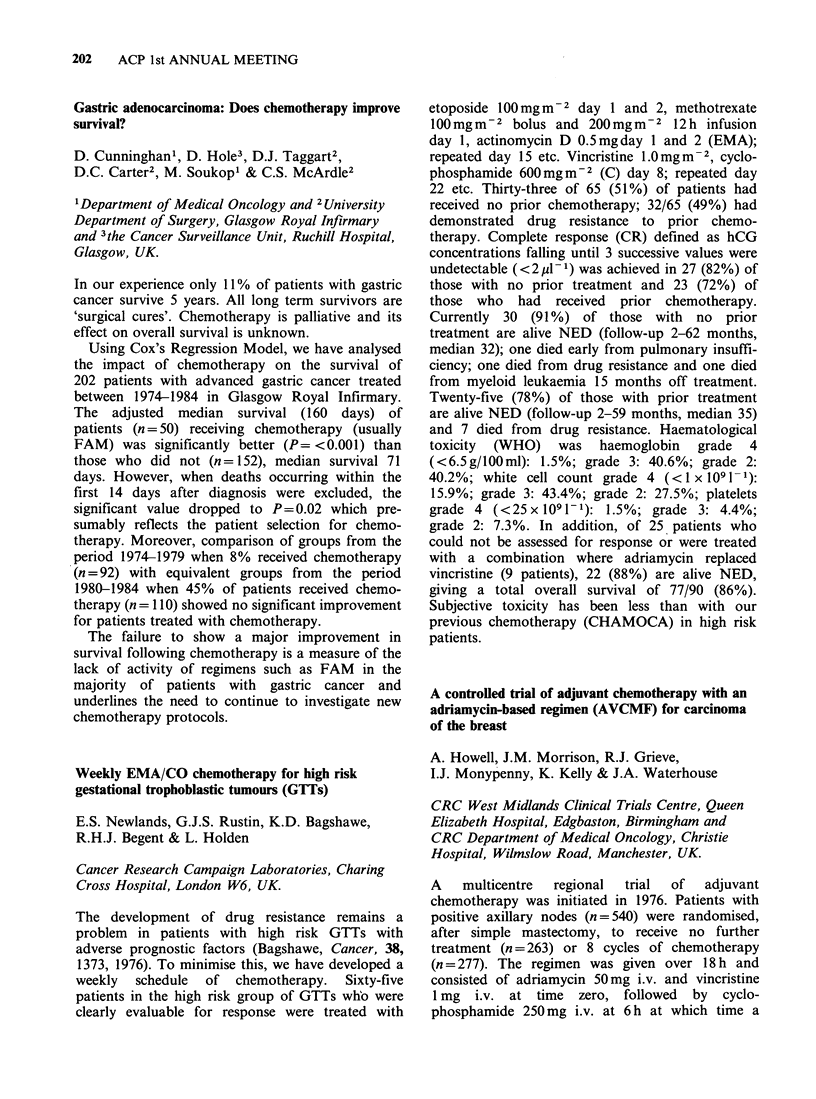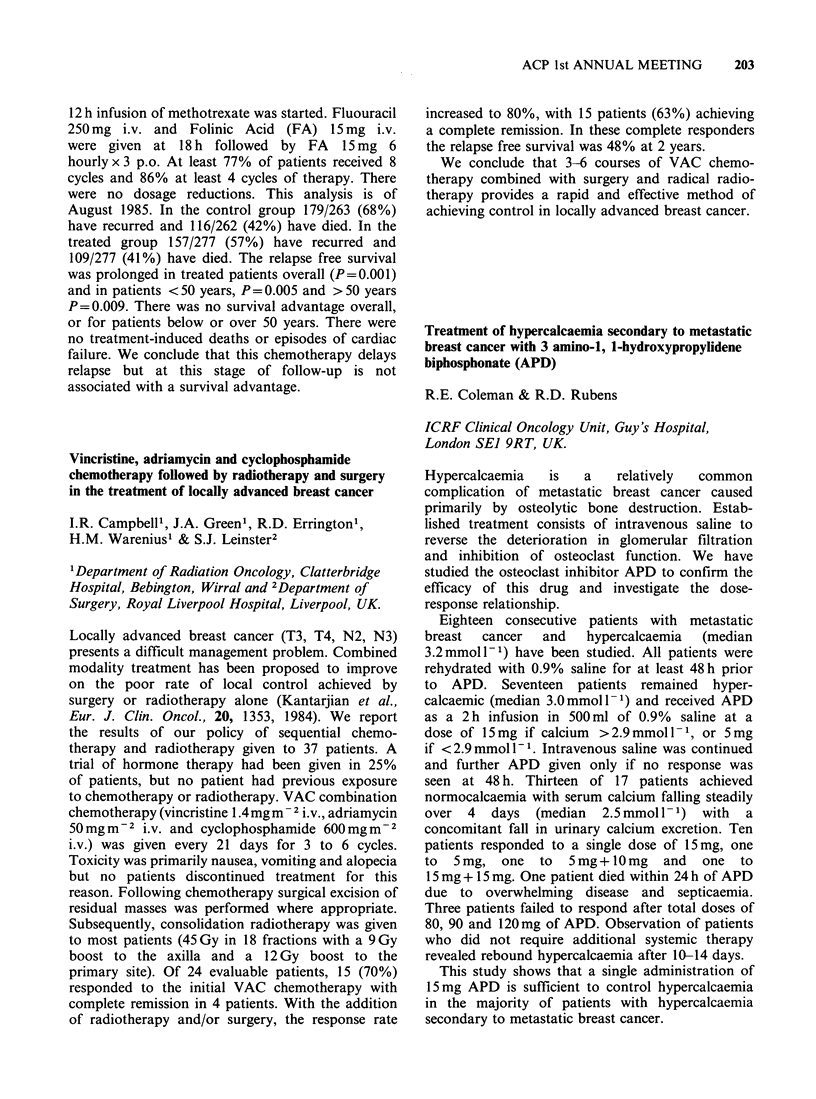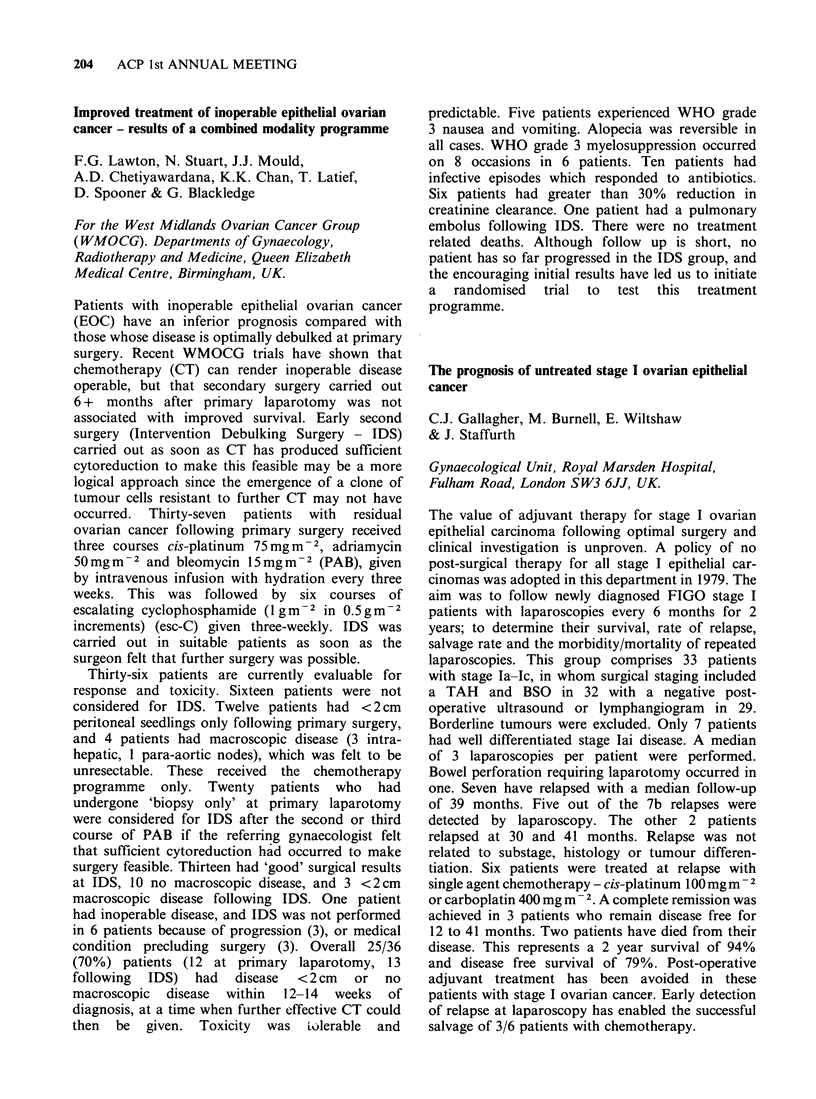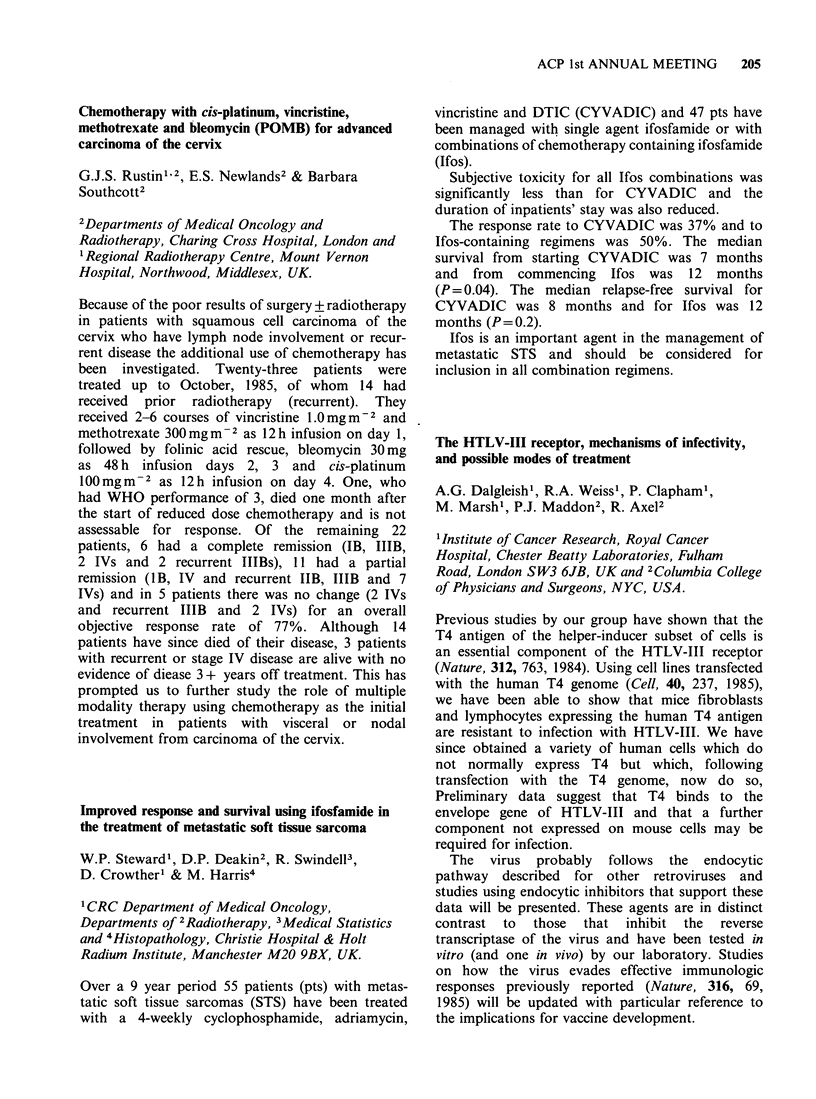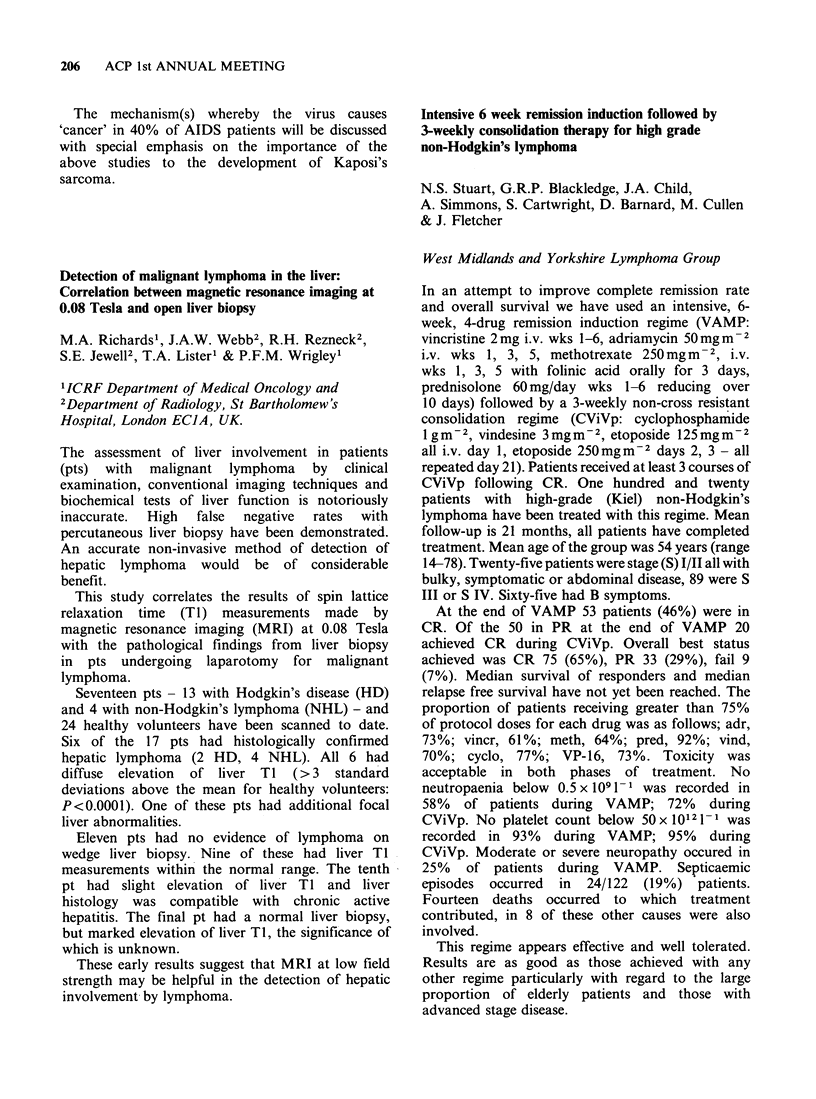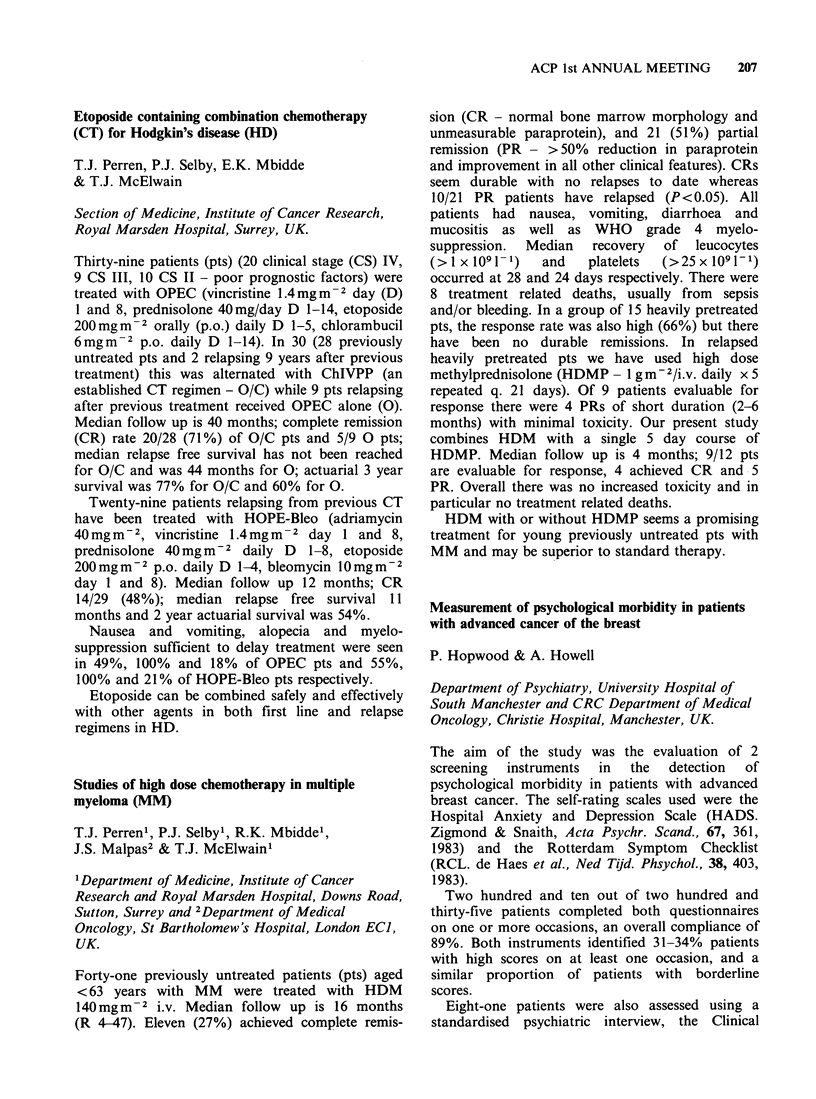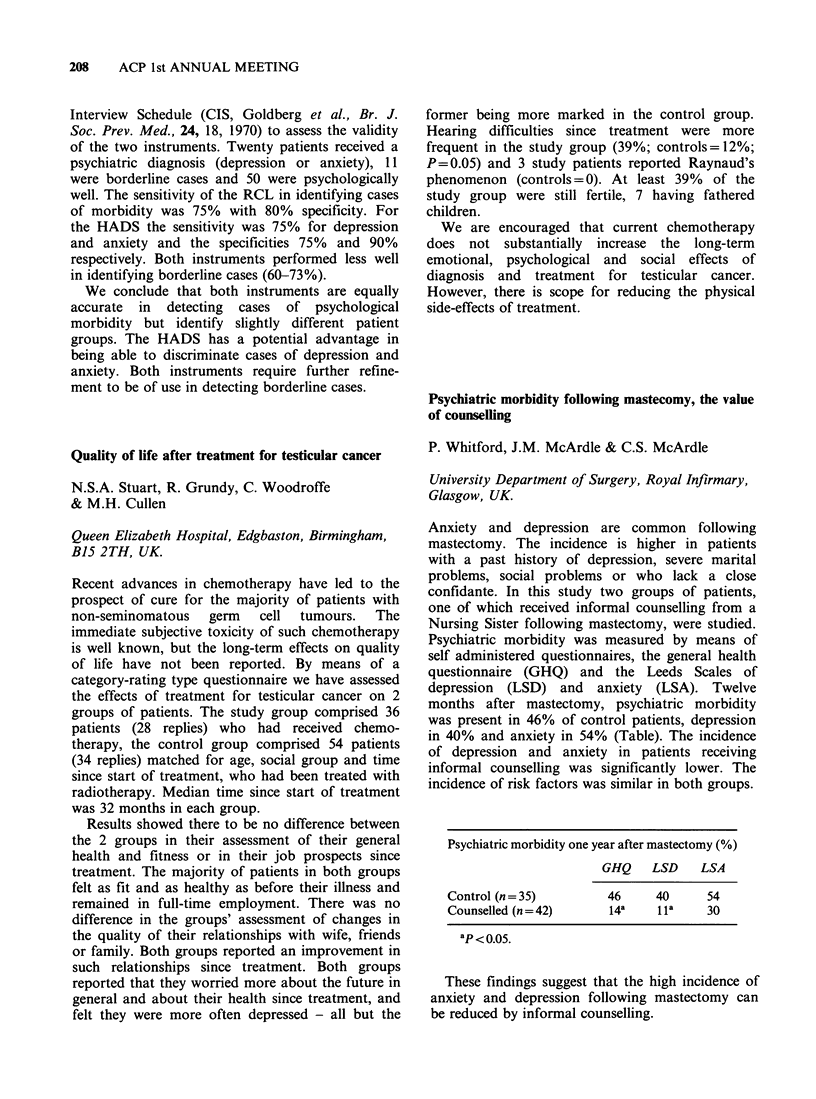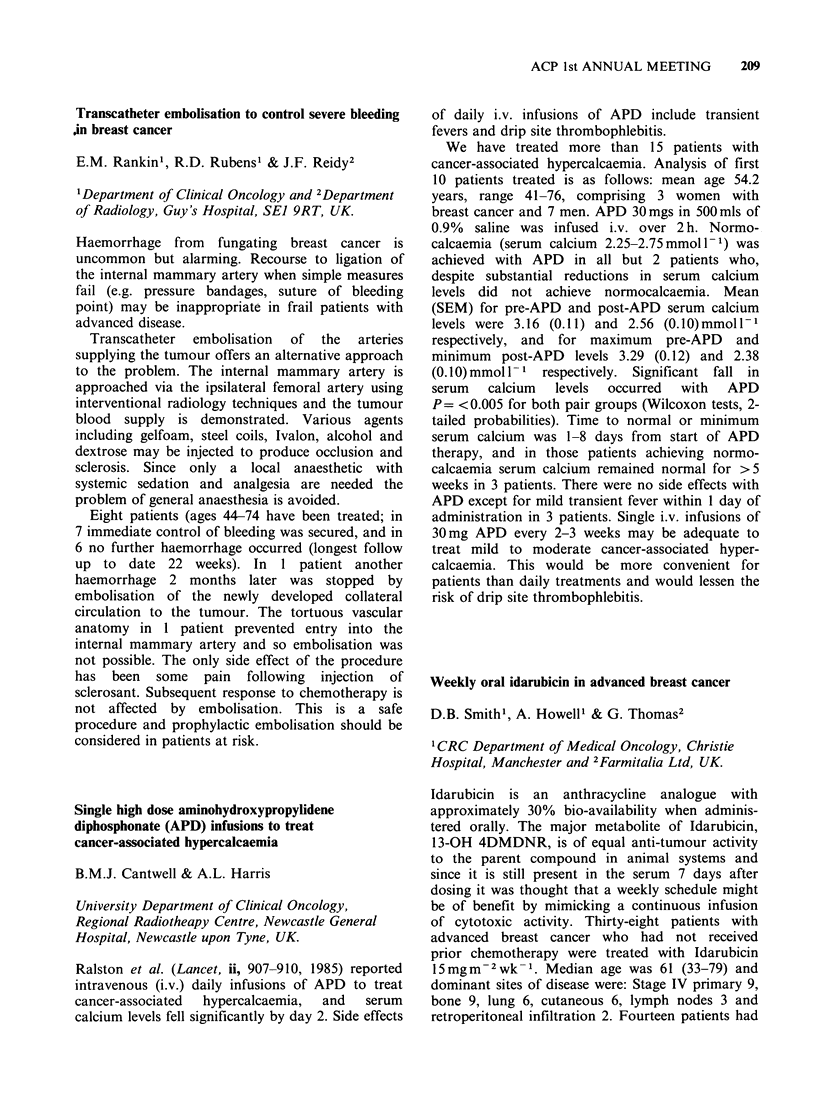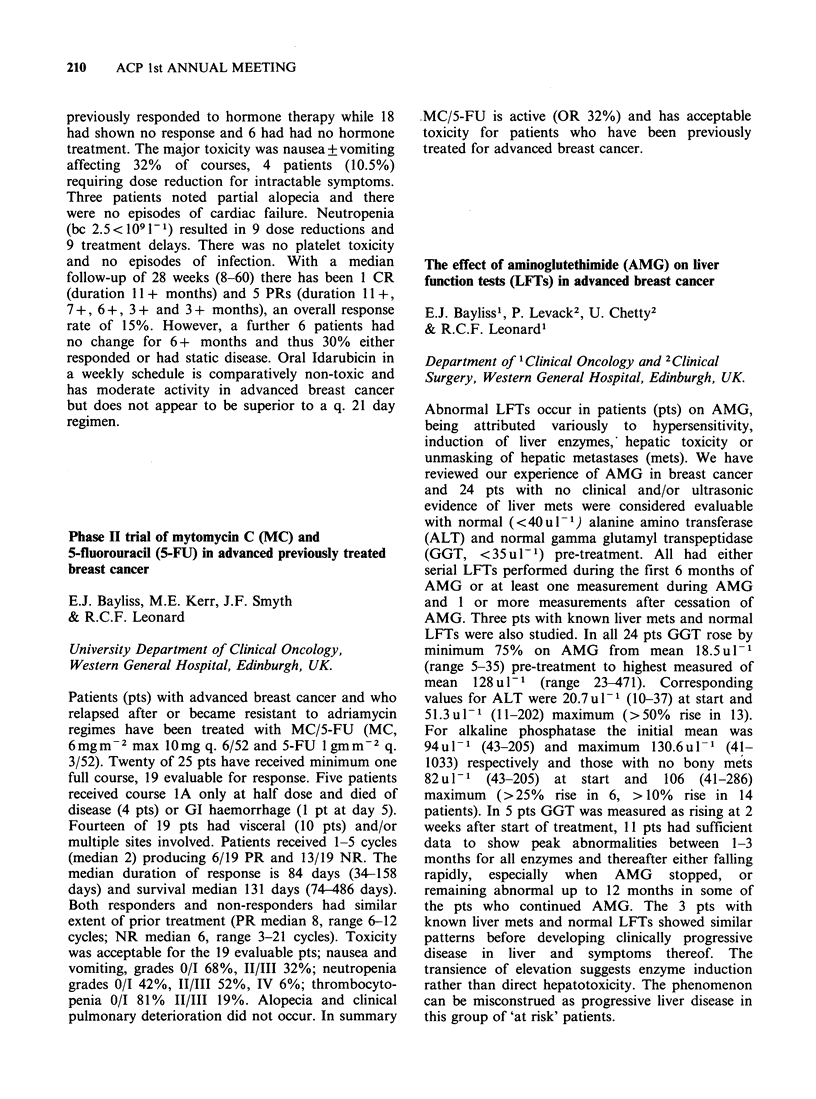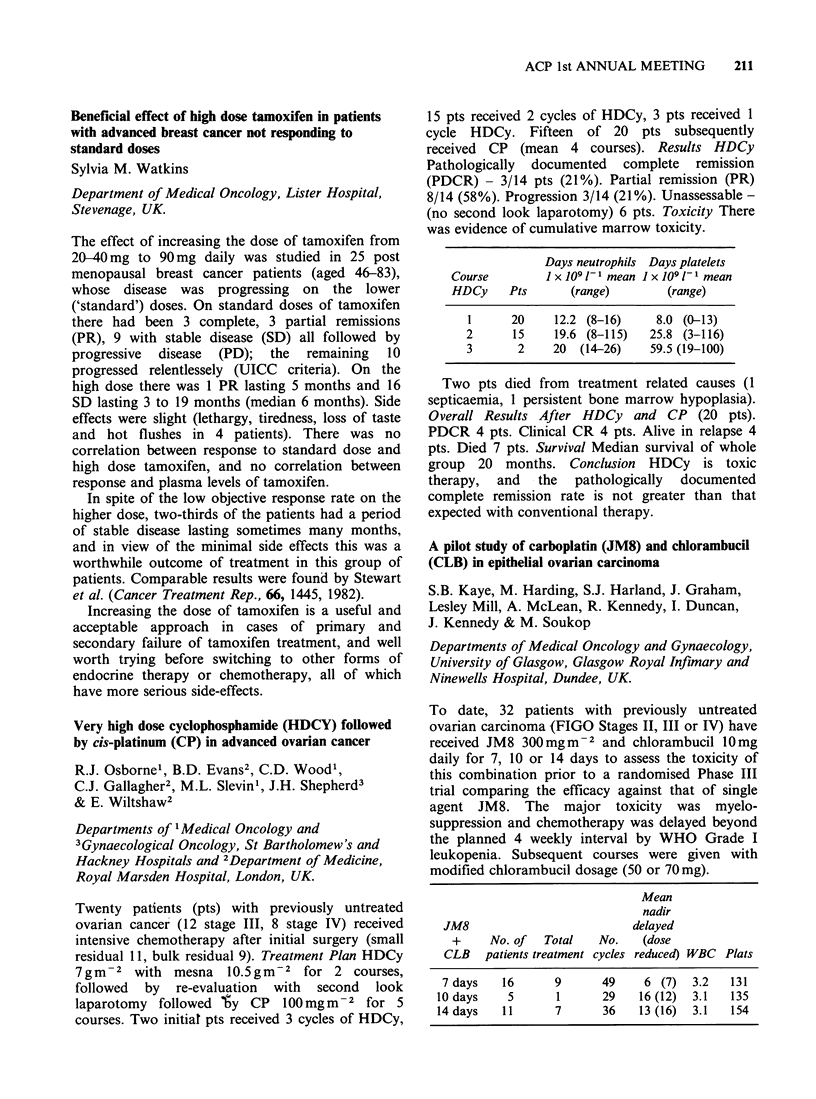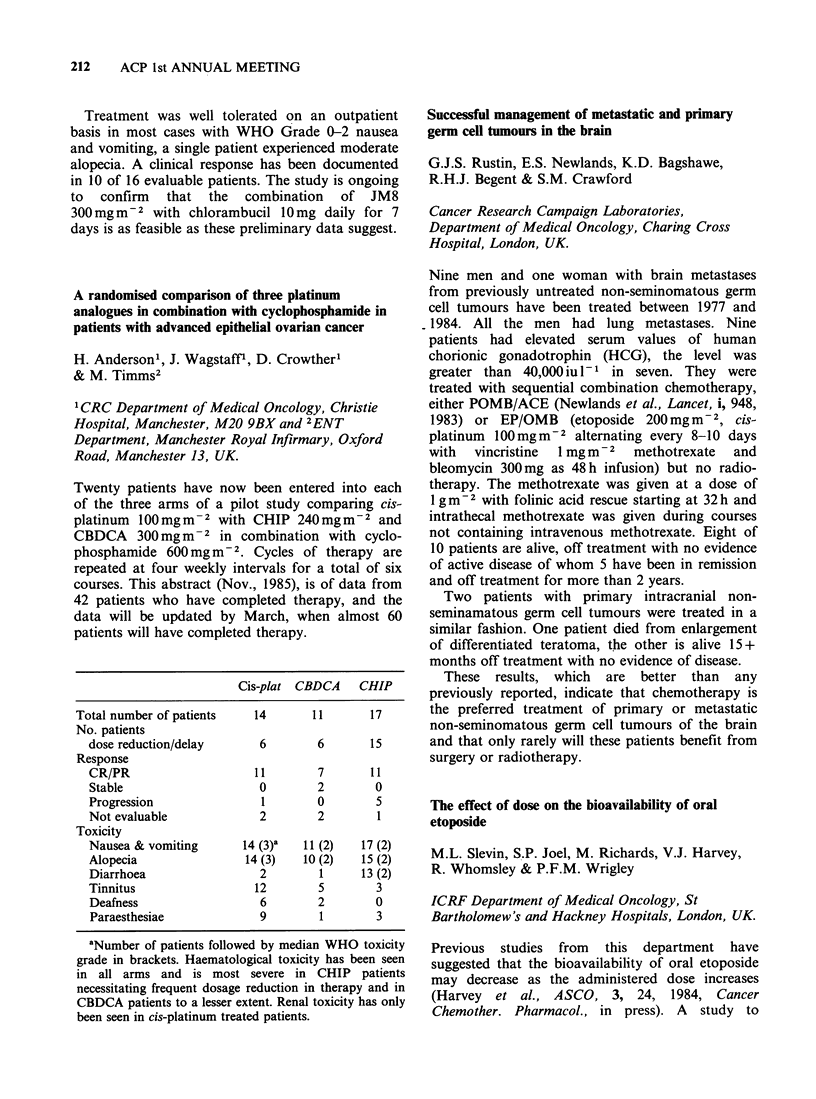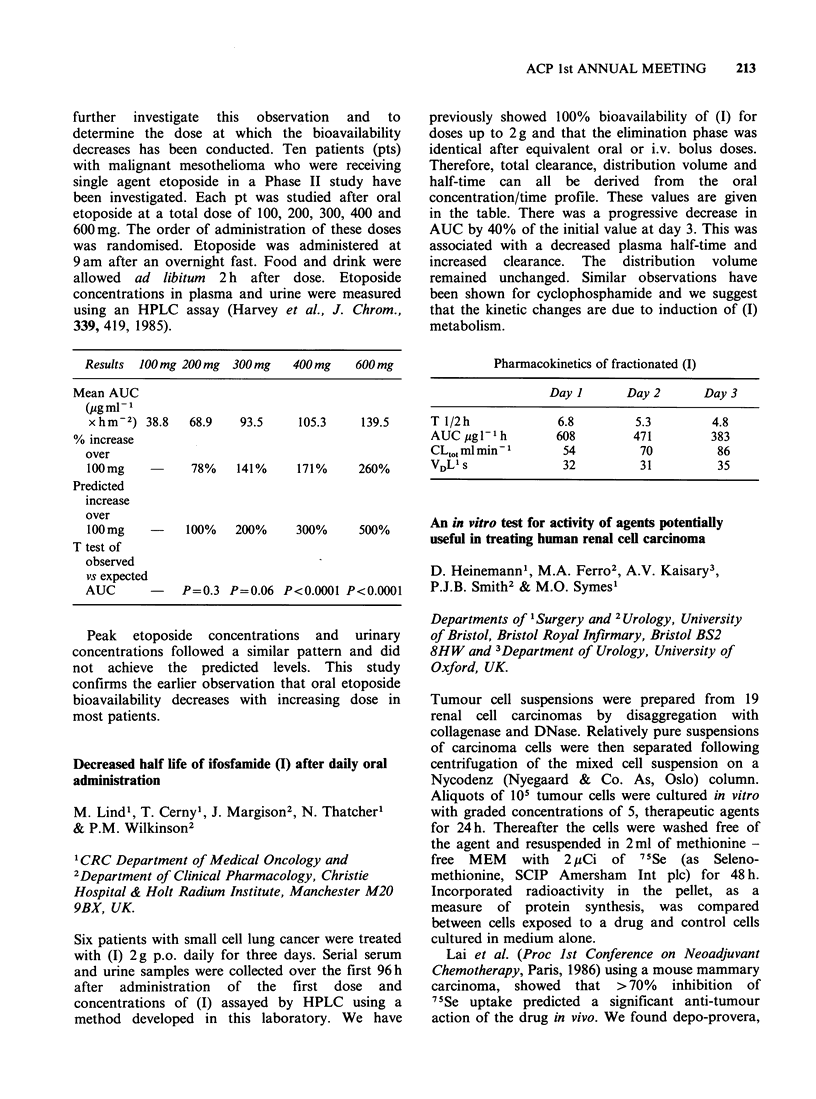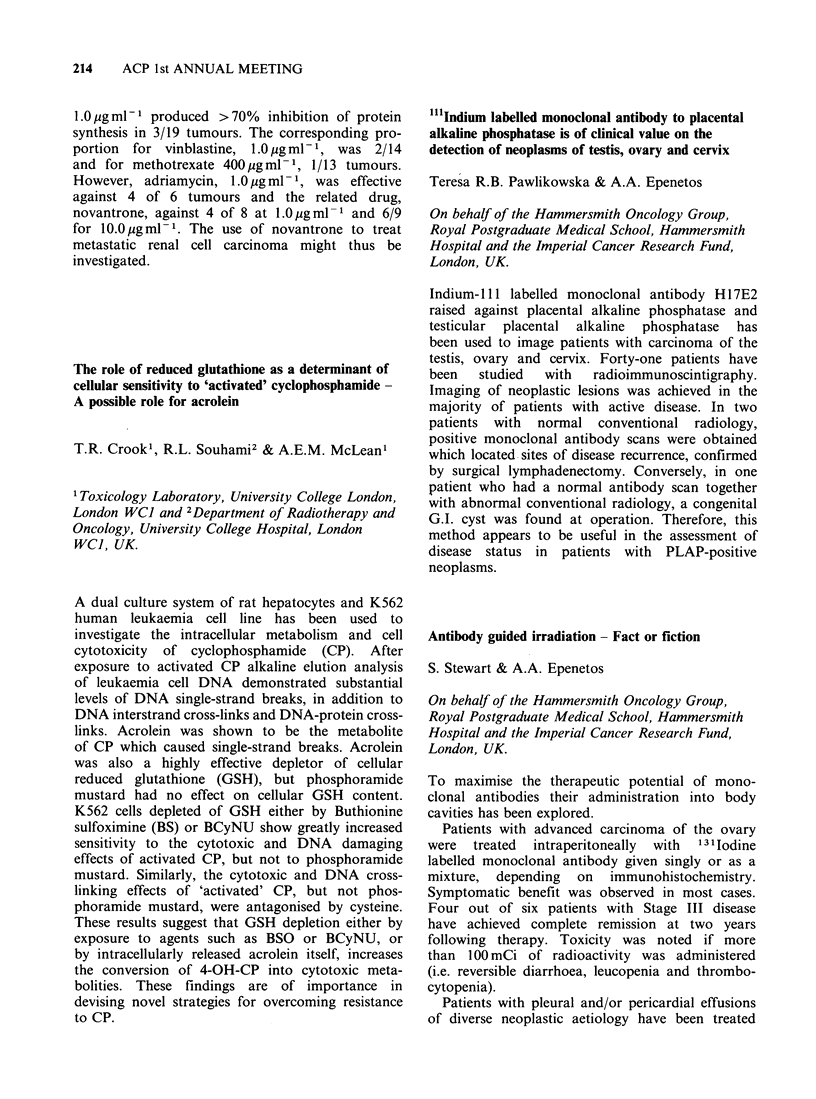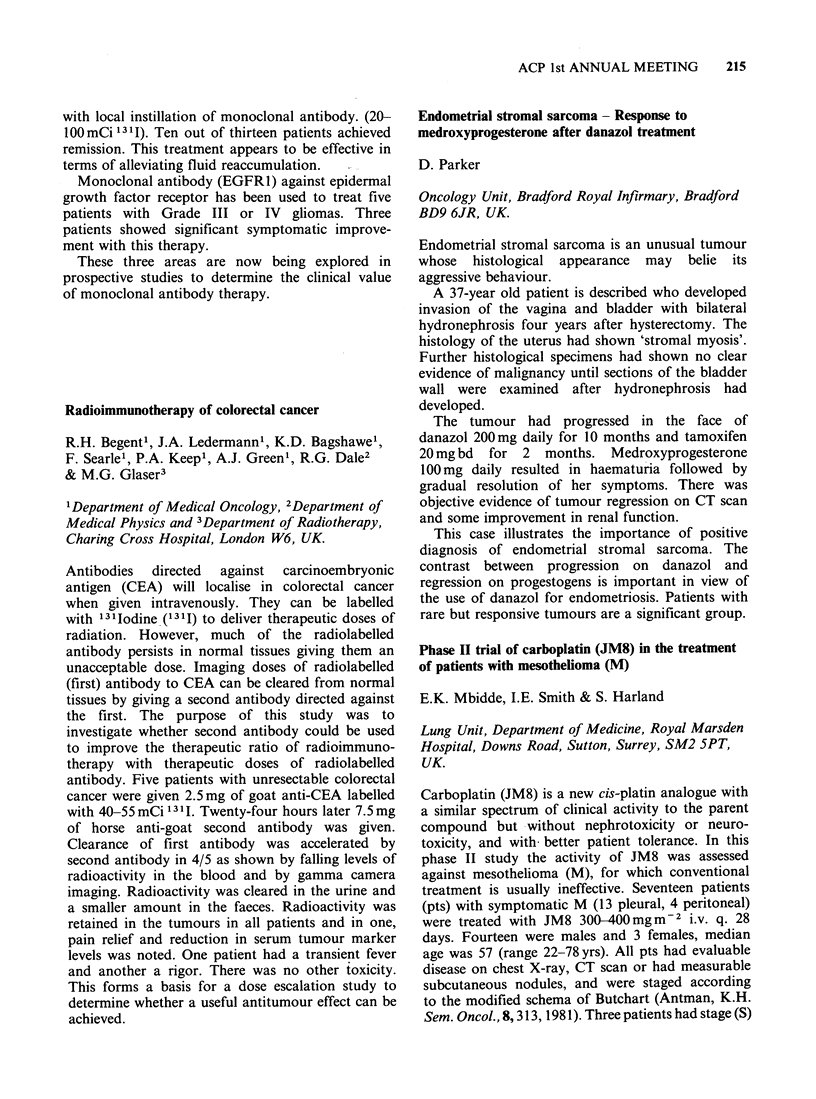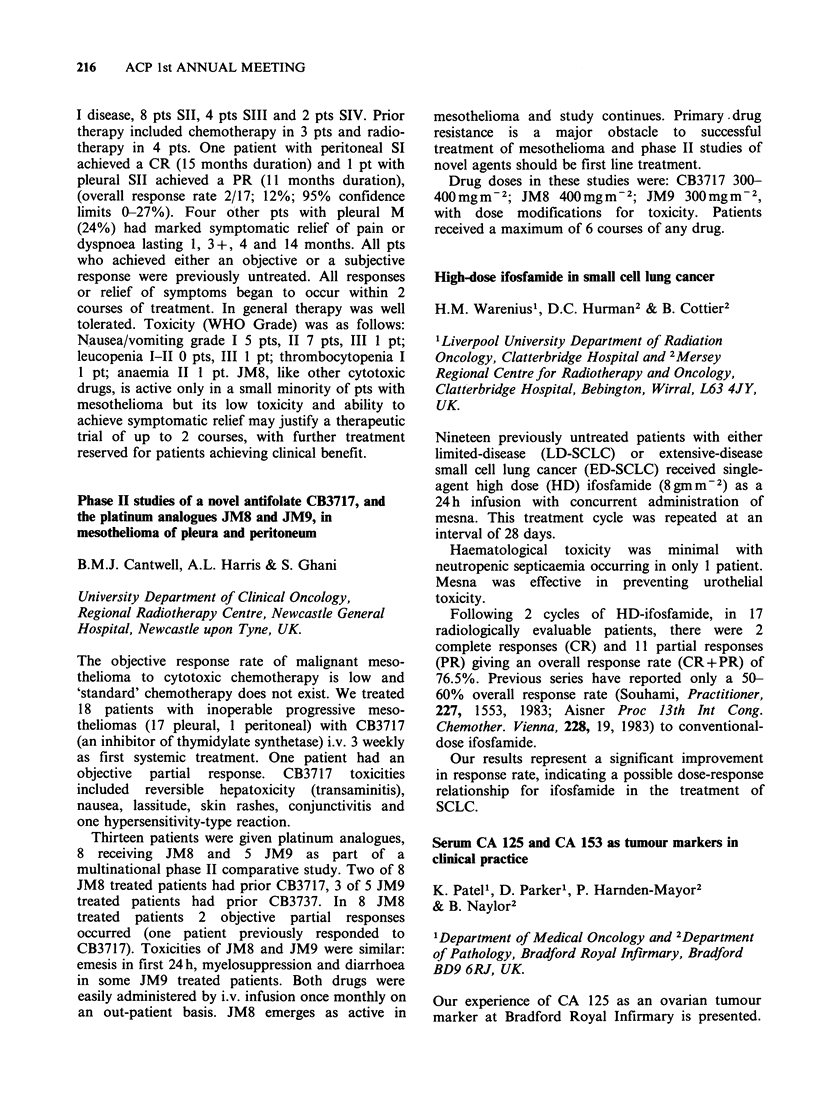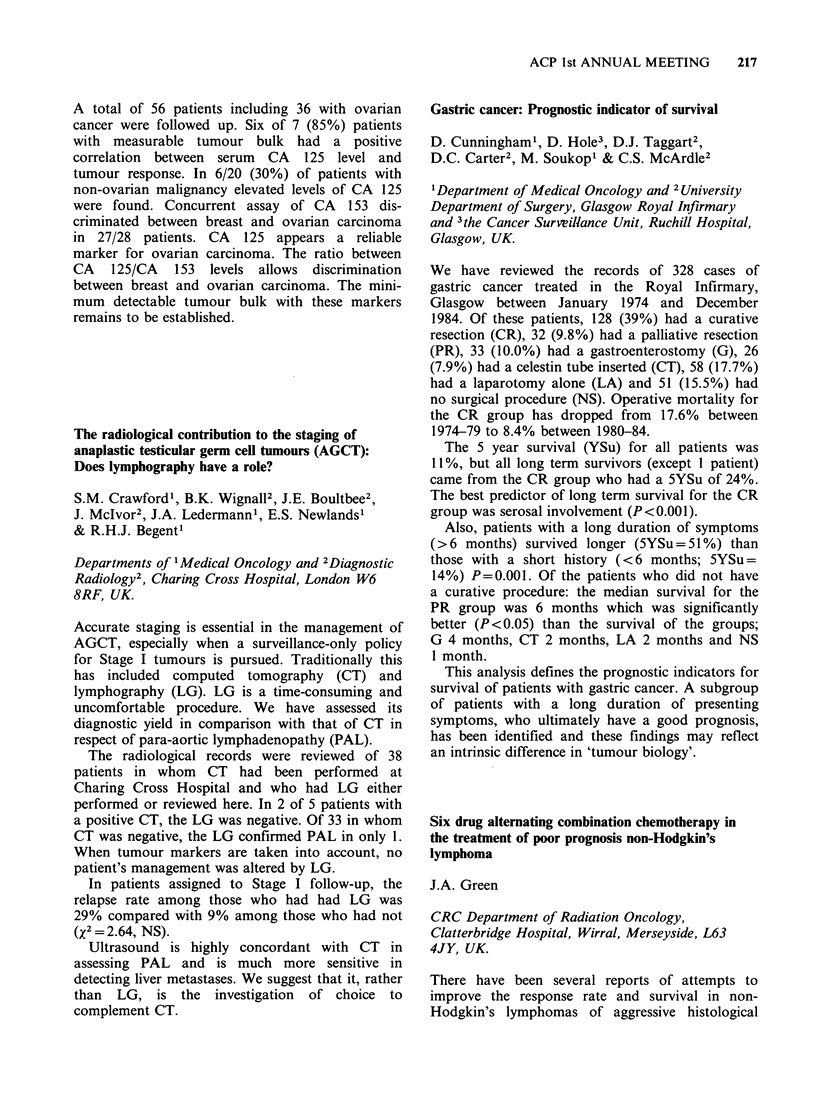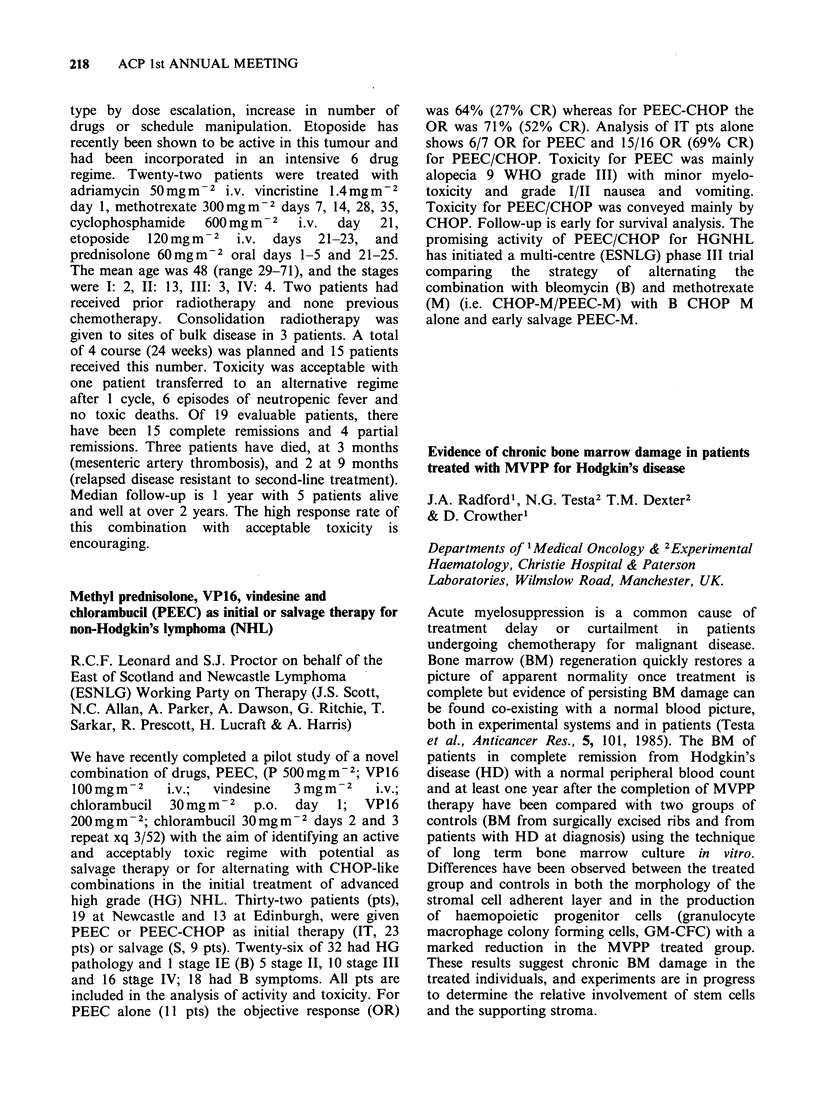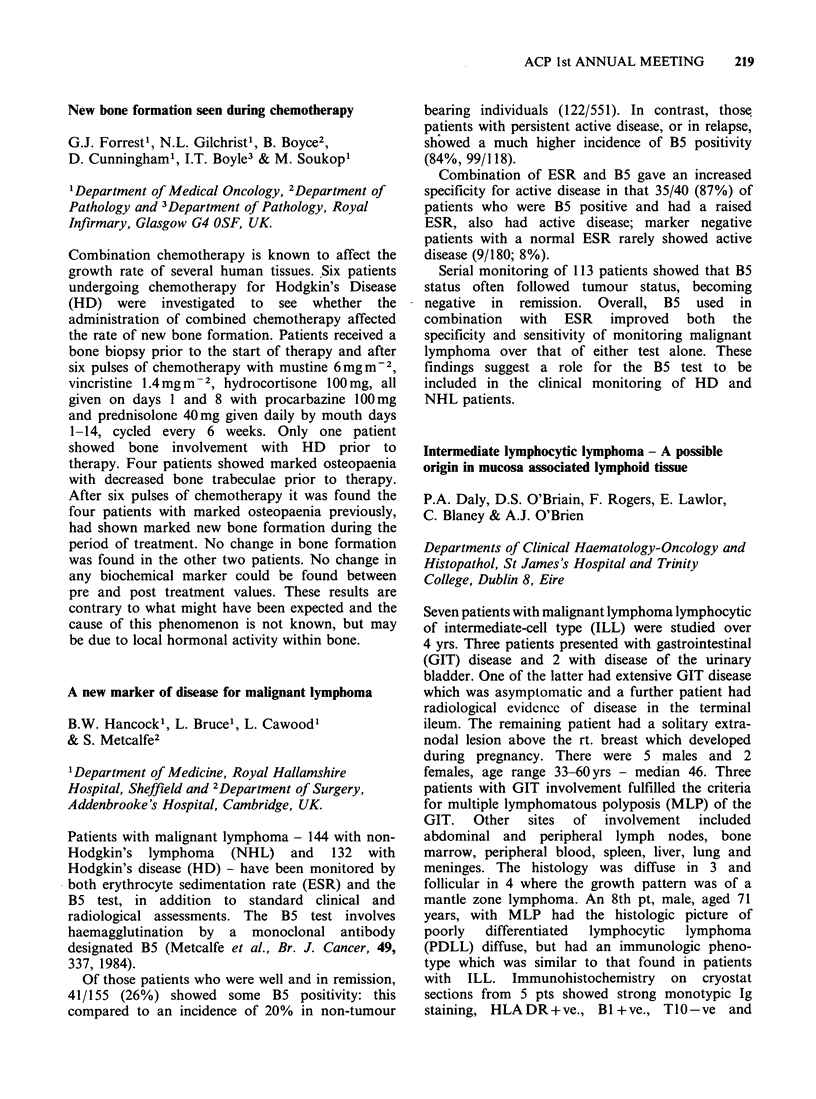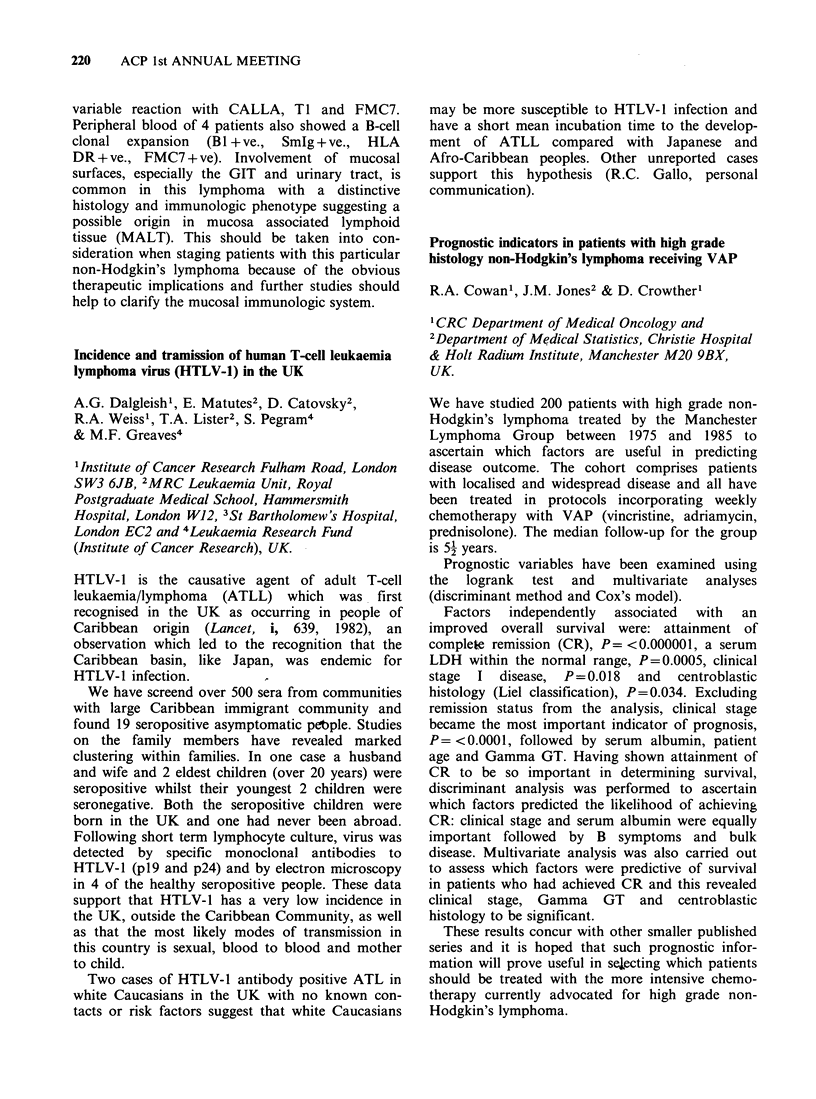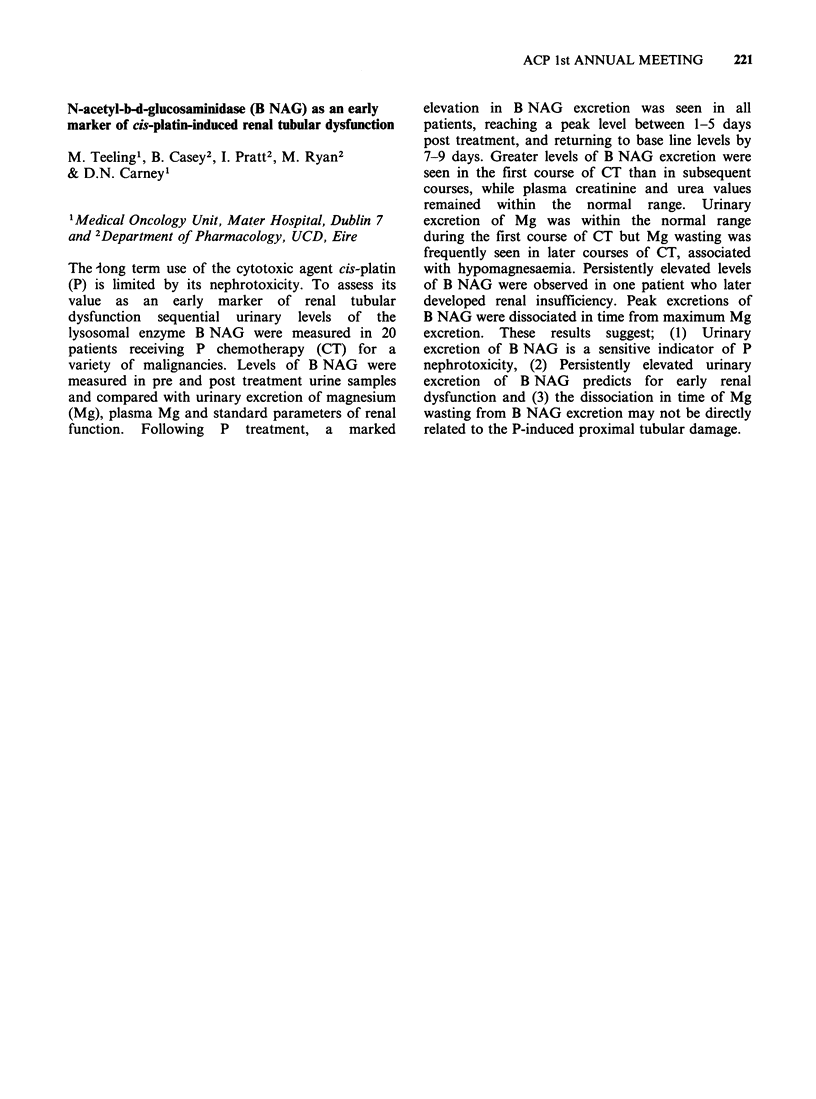# First annual meeting of the Association of Cancer Physicians (in conjunction with the 27th AGM of the British Association for Cancer Research). March 24-26, 1986, Bristol, UK. Abstracts.

**DOI:** 10.1038/bjc.1986.165

**Published:** 1986-07

**Authors:** 


*Br. J. Cancer* (1986), **54**, 199–221

### First Annual Meeting of the Association of Cancer Physicians*

### (In conjunction with the 27th AGM of the British Association for Cancer Research) March 24–25, 1986

*Held at the University of Bristol, UK.*

### Abstracts of members' proffered papers†

### High dose hydroxyurea as a DNA repair inhibitor: A phase I and II study in lung cancer

D. Veale, B.J.M. Cantwell, N. Kerr, A. Upfold, M. Earnshaw & A.L. Harris

*Clinical Oncology and Pharmaceutical Quality Control Laboratory, Newcastle General Hospital, Newcastle upon Tyne, UK.*

Hydroxyurea (HO) is an S-phase cell-cycle-specific agent that selectively inhibits DNA synthesis, by inhibition of ribonucleotide reductase. HO concentrations >1 mM inhibit DNA synthesis by >99% in human lung cancer lines *in vitro*. Concentrations between 1 and 10 mM inhibit DNA repair. We conducted a phase 1 trial to achieve levels >1 mM with the aim of using hydroxyurea as a repair inhibitor and to assess duration for which this could be maintained without marrow and tissue toxicity. Seventeen patients with advanced non-small cell lung cancer were given HO 1 g h$^{-1}$ by i.v. infusion or 6G hourly p.o. to a dose of 24 g/24 h $n=7$, 32 g/32 h $n=1$, 36 g/36 h $n=26$, 48 g/48 h $n=9$. There was no marrow toxicity at 24 h, 2 WHO grade 1, 1 grade 2 at 36 h and 1 grade 3 plus 1 grade 2 at 48 h. Five patients had some nausea and vomiting and 1 had mucositis. Two patients showed evidence of radiological response after three courses of treatment, and three had evidence of static disease. Serum HO levels were monitored in 26 i.v. and in 5 oral courses of treatment. Serum level of >1 mM was maintained from 6 h. There was a small variation in levels achieved (s.e. 5% of the mean area under the curve). Thus we have shown that HO can be given by infusion for 48 h to achieve a serum concentration above 1 mM. This regimen could be used for DNA repair inhibition and potentiation of other drugs

*Enquiries to the ACP Secretariat, Department of Medical Oncology, Christie Hospital and Holt Radium Institute, Manchester M20 9BX, UK.

†Reprints of these abstracts are not available – Editor.

### A randomised trial to evaluate the effect of schedule on the activity of etoposide in small cell lung cancer

M.L. Slevin, P.I. Clark, M. Richards, R.J. Osborne, S. Malik, C.D. Wood, V.J. Harvey, S.P. Joel, J.S. Malpas & P.F.M. Wrigley

*ICRF Department of Medical Oncology, St Bartholomew's and Hackney Hospitals, London, UK.*

The activity of two different schedules of etoposide in small cell lung cancer (SCLC) has been investigated. Forty patients (pts) with previously untreated extensive SCLC were randomised to receive single agent etoposide 500 mg m$^{-2}$ either as a 24 h infusion, or as 5 daily doses of 100 mg m$^{-2}$ given as an infusion over 2 h. It was shown that etoposide is stable in solution for 24 h. Both regimes were repeated every 21 days for 6 cycles. At relapse pts with a Karnofsky performance score of 60 or more received a combination of cyclophosphamide (750 mg m$^{-2}$), vincristine (2 mg) and adriamycin (50 mg m$^{-2}$), whilst those with a score of 50 or less received radiotherapy or other symptomatic treatment. The same therapy was used at relapse in both arms of the study.

Thirty-eight patients are currently evaluable. The two groups were equal as regards Karnofsky performance status, number of metastatic sites, bone marrow involvement, albumin and serum sodium.

| *Results* | *Evaluable patients* | *Partial response* | *Complete response* | *Median survival* |
|---|---|---|---|---|
| 24 h infusions | 20 | 2 (10%) | 0 | 167 days |
| 5 daily doses | 18 | 14 (78%) | 0 | 294 days |

© The Macmillan Press Ltd., 1986

The partial response rate was significantly greater in the 5 day schedule ($P = <0.0001$) as was median survival ($P=0.03$). Treatment was well tolerated with few side effects in either arm. Bone marrow toxicity was mild. Etoposide pharmacokinetics were measured in all pts and the total areas under the curve were equivalent in both arms. This study demonstrates that a 5 day schedule of etoposide is clearly superior to a 24 h schedule in SCLC.

### High dose carboplatin (JM8) in patients with lung cancer: A phase I study

M.E. Gore, A.H. Calvert & I.E. Smith

*Lung Unit, Royal Marsden Hospital, Downs Road, Sutton, Surrey, UK.*

Carboplatin (JM8) is an active analogue of *cis*-platin in small cell lung cancer. Its main advantage is that it is without the nephro-, neuro- and oto-toxicity of its parent compound *cis*-platin. In conventional dosage (400 mg m$^{-2}$ i.v. q. 28 days) the dose limiting toxicity of JM8 is myelosuppression. We have investigated the feasibility of giving high doses of JM8 and have treated 5 patients at 800 mg m$^{-2}$ (9 courses), 4 patients at 1.2 g m$^{-2}$ (5 courses) and 4 patients at 1.6 g m$^{-2}$ (5 courses). Seven patients had small cell lung cancer (SCLC) and 6 had non-small cell lung cancer. Sixteen courses were evaluable and myelosuppression was the major toxicity. At 1.2 g m$^{-2}$ the white cell count (WCC) fell below $2 \times 10^9 l^{-1}$ on day 9 for 7 days, the nadir was $0.8 \times 10^9 l^{-1}$ on day 14, the platelet count fell below $100 \times 10^9 l^{-1}$ on day 8 for 9 days, nadir $17 \times 10^9 l^{-1}$ on day 14. At 1.6 g m$^{-2}$ the WCC fell below $2 \times 10^9 l^{-1}$ on day 8 for 6 days, nadir $0.7 \times 10^9 l^{-1}$ on day 12, and the platelets fell below $100 \times 10^9 l^{-1}$ on day 9 for 11 days, nadir $12 \times 10^9 l^{-1}$ on day 14. Treatment was well tolerated with only 2 patients developing lethargy and malaise for 6 days after treatment at 1.2 g and 1.6 g m$^{-2}$. Significant peripheral neuropathy or oto-toxicity was not seen but 1 patient developed alopecia severe enough to require a wig. Cumulative myelotoxicity did not seem to occur at any dose level. EDTA clearances, however, fell in 5/11 evaluable patients by >25% but never >50%. Five patients with SCLC achieved either complete remission (2) or partial remission (3). High dose JM8 up to 1600 m m$^{-2}$ is feasible, well tolerated, does not require autologous bone marrow transplantation and appears very active against SCLC.

### A new thymidylate synthase (TS) inhibitor, CB3717, in human primary liver cancer (PLC): *In vitro*, xenograft and phase II studies

M.F. Bassendine, A.L. Harris, N. Curtin & O. James

*Departments of Medicine, Clinical Oncology & Cancer Research Unit, University of Newcastle upon Tyne, UK.*

PLC is the commonest tumour world-wide but there is a lack of effective drugs. Because of high TS activity and rapid thymidine breakdown in some hepatomas, TS is a rational target for therapy. We have investigated a new TS inhibitor, CB3717, in 2 human PLC cell lines (PLC/PRF/5, HEP3B/NU71) *in vitro*, the $ID_{50}$ for CB3717 was 2–3 $\mu$m, a level of achieved *in vivo* with 300 mg m$^{-2}$ i.v. The lines were grown as xenografts and both were inhibited by CB3717. These lines appeared more sensitive than some other xenografts. The activity of CB3717 led to a phase II trial. Fourteen patients with histologically proven PLC were treated with 300 mg m$^{-2}$ i.v. every 3 weeks. There were 8 men, 6 women, age 27–74 years, mean 56 years; 11 had cirrhosis. Six were grade A (expected survival > 14/52), 8 were grade B (expected survival < 14/52). There were 6 responders with > 50% reduction in FP and 3 of these had greater than a 1 log fall in FP. Five of 6 responders were female. The median survival of non-responders was 1 month, responders 13 months, 4 still alive. TS inhibition is an effective target in hepatoma and ways to enhance this are being studied.

### The effect of verapamil on the pharmacokinetics of adriamycin

D. Kerr[1], J. Graham[1], J. Cummings[1], G. Morrison[1], M.J. Brodie[2] & S.B. Kaye[1]

*Departments of [1]Medical Oncology and [2]Medicine, University of Glasgow, Glasgow, UK.*

Evidence from a wide range of experimental tumour systems indicates that the calcium channel blocker verapamil can enhance the cytotoxic efficacy of adriamycin, thereby circumventing resistance to this drug, and several phase I clinical studies of the combination of the 2 drugs have been initiated. Since adriamycin undergoes extensive hepatic metabolism, and verapamil increases hepatic blood flow and inhibits the hepatic microsomal enzyme system, there is the potential for a major pharmacological interaction between these 2 drugs in clinical practice. To investigate this we have examined the influence of verapamil on the kinetics of adriamycin in patients with small cell lung cancer. Five patients were treated with combination chemotherapy comprising adriamycin, cyclophosphamide, vincristine and VP16. Oral verapamil (80 mg TDS for 3 days followed by 120 mg QDS for 4 days) was given with one or other of the first 2 courses of chemotherapy, in random order. Adriamycin and verapamil were measured by sensitive and specific HPLC techniques based on fluorescence detection. Combined treatment with verapamil increased peak levels of adriamycin ($2289 \pm 1243$ *vs* $1122 \pm 898\,\mathrm{ng\,ml^{-1}}$), the terminal 1/2-life ($36 \pm 12$ *vs* $23.2 \pm 6.8\,\mathrm{h}$), and steady state vol. of distribution ($1360 \pm 950$ *vs* $998 \pm 1000\,\mathrm{l}$), whereas the vol. of central compartment ($16.8 \pm 14$ *vs* $31.4 \pm 25\,\mathrm{l}$) and plasma clearance ($36 \pm 18$ *vs* $46 \pm 16\,\mathrm{l\,h^{-1}}$) were reduced. Adriamycinol and 7-deoxyaglycone metabolite levels were similar in both groups. Steady state verapamil levels ranged from 80–110 $\mathrm{ng\,ml^{-1}}$. It would appear from this preliminary study that there is a significant pharmacokinetic interaction between adriamycin and verapamil which could result in enhanced activity.

### Nabilone, prochlorperazine and dexamethasone – good antiemetic control with few side effects

G.J. Forrest[1], D. Cunningham[1], A. Hutcheon[2], R. Moss[1], T. Young[1] & M. Soukop[1]

[1]*Department of Medical Oncology, Royal Infirmary, Glasgow and* [2]*Department of Clinical Oncology, Royal Infirmary, Aberdeen, UK.*

Our previous experience has shown nabilone (2 mg) and prochlorperazine (5 mg) to be a useful combination in controlling chemotherapy induced emesis. However, at these doses drowsiness and xerostomia were quite frequent and dysphoria occurred occasionally. We have, therefore, performed a double blind crossover study comparing nabilone 1 mg (N) and prochlorperazine 5 mg (P) given at least 2 h prior to therapy and again at 10 pm on the evening of therapy, with N, P and 20 mg dexamethasone (D) given i.v. with therapy. Sixty-three patients receiving chemotherapy without *cis*-platin have entered the study with 57 patients completing the crossover. With N+P+D there was complete control of vomiting in 67% of patients which was significantly better ($P < 0.01$) than N+P alone. Side effects were infrequent or mild and only 5% of patients experience severe sedation. No patient reported dysphoria. There was a significant patient preference ($P < 0.01$) for the dexamethasone containing arm.

This study demonstrates that dexamethasone contributes significantly to the emetic control of nabilone and prochlorperazine, and has also confirmed the abolition of CNS side effects of nabilone when this drug is combined with prochlorperazine.

### Fertility after chemotherapy for male and female germ cell tumours

D. Pektasides, G.J.S. Rustin, K.D. Bagshawe, E.S. Newlands & R.H.J. Begent

*Department of Medical Oncology, Cancer Research Campaign Laboratories, Charing Cross Hospital, London W6, UK.*

Gonadal function was assessed in 59 men and 31 women who successfully completed chemotherapy with the POMB/ACE regimen (Newlands *et al.*, *Lancet*, **i**, 948, 1983) for germ cell tumours. Spermatogenesis had recovered in 29 (49%) of the men from 24–84 months after completion of treatment. None of the 11 men who received para-aortic radiotherapy in addition to chemotherapy recovered spermatogenesis. Other factors that were associated with permanent sterility included original tumour bulk $>5\,\mathrm{cm}$ diameter ($P = 0.011$), and duration of chemotherapy $\geq 6$ months ($P = 0.029$). Seventeen (81%) of the 21 patients without these adverse factors recovered spermatogenesis compared with 12 (32%) of 38 patients who had one or more of these factors. There was no significant correlation between azoospermia or oligospermia prior to chemotherapy, age $\geq 25$ or $\geq 30$, or laparotomy during treatment, and recovery of spermatogenesis.

Menstruation was not expected, at the time of analysis, after chemotherapy in 15 of the 31 women because of the extent of surgery in 11, young age in 3, pelvic radiotherapy in 1, 46xy karyotype in 1 and only 3 month period off chemotherapy in 1. All the remaining women whose median age at start of chemotherapy was 21 years (9–38) are now menstruating. Three of these women have so far had successful pregnancies and 9 of the men have fathered children. There have been no congenital abnormalities.

### Gastric adenocarcinoma: Does chemotherapy improve survival?

D. Cunninghan[1], D. Hole[3], D.J. Taggart[2], D.C. Carter[2], M. Soukop[1] & C.S. McArdle[2]

[1]*Department of Medical Oncology and* [2]*University Department of Surgery, Glasgow Royal Infirmary and* [3]*the Cancer Surveillance Unit, Ruchill Hospital, Glasgow, UK.*

In our experience only 11% of patients with gastric cancer survive 5 years. All long term survivors are 'surgical cures'. Chemotherapy is palliative and its effect on overall survival is unknown.

Using Cox's Regression Model, we have analysed the impact of chemotherapy on the survival of 202 patients with advanced gastric cancer treated between 1974–1984 in Glasgow Royal Infirmary. The adjusted median survival (160 days) of patients ($n=50$) receiving chemotherapy (usually FAM) was significantly better ($P=<0.001$) than those who did not ($n=152$), median survival 71 days. However, when deaths occurring within the first 14 days after diagnosis were excluded, the significant value dropped to $P=0.02$ which presumably reflects the patient selection for chemotherapy. Moreover, comparison of groups from the period 1974–1979 when 8% received chemotherapy ($n=92$) with equivalent groups from the period 1980–1984 when 45% of patients received chemotherapy ($n=110$) showed no significant improvement for patients treated with chemotherapy.

The failure to show a major improvement in survival following chemotherapy is a measure of the lack of activity of regimens such as FAM in the majority of patients with gastric cancer and underlines the need to continue to investigate new chemotherapy protocols.

### Weekly EMA/CO chemotherapy for high risk gestational trophoblastic tumours (GTTs)

E.S. Newlands, G.J.S. Rustin, K.D. Bagshawe, R.H.J. Begent & L. Holden

*Cancer Research Campaign Laboratories, Charing Cross Hospital, London W6, UK.*

The development of drug resistance remains a problem in patients with high risk GTTs with adverse prognostic factors (Bagshawe, *Cancer*, **38**, 1373, 1976). To minimise this, we have developed a weekly schedule of chemotherapy. Sixty-five patients in the high risk group of GTTs who were clearly evaluable for response were treated with etoposide $100\,mg\,m^{-2}$ day 1 and 2, methotrexate $100\,mg\,m^{-2}$ bolus and $200\,mg\,m^{-2}$ 12 h infusion day 1, actinomycin D 0.5 mg day 1 and 2 (EMA); repeated day 15 etc. Vincristine $1.0\,mg\,m^{-2}$, cyclophosphamide $600\,mg\,m^{-2}$ (C) day 8; repeated day 22 etc. Thirty-three of 65 (51%) of patients had received no prior chemotherapy; 32/65 (49%) had demonstrated drug resistance to prior chemotherapy. Complete response (CR) defined as hCG concentrations falling until 3 successive values were undetectable ($<2\,\mu l^{-1}$) was achieved in 27 (82%) of those with no prior treatment and 23 (72%) of those who had received prior chemotherapy. Currently 30 (91%) of those with no prior treatment are alive NED (follow-up 2–62 months, median 32); one died early from pulmonary insufficiency; one died from drug resistance and one died from myeloid leukaemia 15 months off treatment. Twenty-five (78%) of those with prior treatment are alive NED (follow-up 2–59 months, median 35) and 7 died from drug resistance. Haematological toxicity (WHO) was haemoglobin grade 4 ($<6.5\,g/100\,ml$): 1.5%; grade 3: 40.6%; grade 2: 40.2%; white cell count grade 4 ($<1\times10^9\,l^{-1}$): 15.9%; grade 3: 43.4%; grade 2: 27.5%; platelets grade 4 ($<25\times10^9\,l^{-1}$): 1.5%; grade 3: 4.4%; grade 2: 7.3%. In addition, of 25 patients who could not be assessed for response or were treated with a combination where adriamycin replaced vincristine (9 patients), 22 (88%) are alive NED, giving a total overall survival of 77/90 (86%). Subjective toxicity has been less than with our previous chemotherapy (CHAMOCA) in high risk patients.

### A controlled trial of adjuvant chemotherapy with an adriamycin-based regimen (AVCMF) for carcinoma of the breast

A. Howell, J.M. Morrison, R.J. Grieve, I.J. Monypenny, K. Kelly & J.A. Waterhouse

*CRC West Midlands Clinical Trials Centre, Queen Elizabeth Hospital, Edgbaston, Birmingham and CRC Department of Medical Oncology, Christie Hospital, Wilmslow Road, Manchester, UK.*

A multicentre regional trial of adjuvant chemotherapy was initiated in 1976. Patients with positive axillary nodes ($n=540$) were randomised, after simple mastectomy, to receive no further treatment ($n=263$) or 8 cycles of chemotherapy ($n=277$). The regimen was given over 18 h and consisted of adriamycin 50 mg i.v. and vincristine 1 mg i.v. at time zero, followed by cyclophosphamide 250 mg i.v. at 6 h at which time a

12 h infusion of methotrexate was started. Fluouracil 250 mg i.v. and Folinic Acid (FA) 15 mg i.v. were given at 18 h followed by FA 15 mg 6 hourly × 3 p.o. At least 77% of patients received 8 cycles and 86% at least 4 cycles of therapy. There were no dosage reductions. This analysis is of August 1985. In the control group 179/263 (68%) have recurred and 116/262 (42%) have died. In the treated group 157/277 (57%) have recurred and 109/277 (41%) have died. The relapse free survival was prolonged in treated patients overall ($P = 0.001$) and in patients $<50$ years, $P = 0.005$ and $>50$ years $P = 0.009$. There was no survival advantage overall, or for patients below or over 50 years. There were no treatment-induced deaths or episodes of cardiac failure. We conclude that this chemotherapy delays relapse but at this stage of follow-up is not associated with a survival advantage.

### Vincristine, adriamycin and cyclophosphamide chemotherapy followed by radiotherapy and surgery in the treatment of locally advanced breast cancer

I.R. Campbell[1], J.A. Green[1], R.D. Errington[1], H.M. Warenius[1] & S.J. Leinster[2]

[1]*Department of Radiation Oncology, Clatterbridge Hospital, Bebington, Wirral and* [2]*Department of Surgery, Royal Liverpool Hospital, Liverpool, UK.*

Locally advanced breast cancer (T3, T4, N2, N3) presents a difficult management problem. Combined modality treatment has been proposed to improve on the poor rate of local control achieved by surgery or radiotherapy alone (Kantarjian *et al.*, *Eur. J. Clin. Oncol.*, **20**, 1353, 1984). We report the results of our policy of sequential chemotherapy and radiotherapy given to 37 patients. A trial of hormone therapy had been given in 25% of patients, but no patient had previous exposure to chemotherapy or radiotherapy. VAC combination chemotherapy (vincristine 1.4 $mg\,m^{-2}$ i.v., adriamycin 50 $mg\,m^{-2}$ i.v. and cyclophosphamide 600 $mg\,m^{-2}$ i.v.) was given every 21 days for 3 to 6 cycles. Toxicity was primarily nausea, vomiting and alopecia but no patients discontinued treatment for this reason. Following chemotherapy surgical excision of residual masses was performed where appropriate. Subsequently, consolidation radiotherapy was given to most patients (45 Gy in 18 fractions with a 9 Gy boost to the axilla and a 12 Gy boost to the primary site). Of 24 evaluable patients, 15 (70%) responded to the initial VAC chemotherapy with complete remission in 4 patients. With the addition of radiotherapy and/or surgery, the response rate increased to 80%, with 15 patients (63%) achieving a complete remission. In these complete responders the relapse free survival was 48% at 2 years.

We conclude that 3–6 courses of VAC chemotherapy combined with surgery and radical radiotherapy provides a rapid and effective method of achieving control in locally advanced breast cancer.

### Treatment of hypercalcaemia secondary to metastatic breast cancer with 3 amino-1, 1-hydroxypropylidene biphosphonate (APD)

R.E. Coleman & R.D. Rubens

*ICRF Clinical Oncology Unit, Guy's Hospital, London SE1 9RT, UK.*

Hypercalcaemia is a relatively common complication of metastatic breast cancer caused primarily by osteolytic bone destruction. Established treatment consists of intravenous saline to reverse the deterioration in glomerular filtration and inhibition of osteoclast function. We have studied the osteoclast inhibitor APD to confirm the efficacy of this drug and investigate the dose-response relationship.

Eighteen consecutive patients with metastatic breast cancer and hypercalcaemia (median 3.2 $mmol\,l^{-1}$) have been studied. All patients were rehydrated with 0.9% saline for at least 48 h prior to APD. Seventeen patients remained hypercalcaemic (median 3.0 $mmol\,l^{-1}$) and received APD as a 2 h infusion in 500 ml of 0.9% saline at a dose of 15 mg if calcium $>2.9\,mmol\,l^{-1}$, or 5 mg if $<2.9\,mmol\,l^{-1}$. Intravenous saline was continued and further APD given only if no response was seen at 48 h. Thirteen of 17 patients achieved normocalcaemia with serum calcium falling steadily over 4 days (median 2.5 $mmol\,l^{-1}$) with a concomitant fall in urinary calcium excretion. Ten patients responded to a single dose of 15 mg, one to 5 mg, one to 5 mg + 10 mg and one to 15 mg + 15 mg. One patient died within 24 h of APD due to overwhelming disease and septicaemia. Three patients failed to respond after total doses of 80, 90 and 120 mg of APD. Observation of patients who did not require additional systemic therapy revealed rebound hypercalcaemia after 10–14 days.

This study shows that a single administration of 15 mg APD is sufficient to control hypercalcaemia in the majority of patients with hypercalcaemia secondary to metastatic breast cancer.

**Improved treatment of inoperable epithelial ovarian cancer – results of a combined modality programme**

F.G. Lawton, N. Stuart, J.J. Mould,
A.D. Chetiyawardana, K.K. Chan, T. Latief,
D. Spooner & G. Blackledge

*For the West Midlands Ovarian Cancer Group (WMOCG). Departments of Gynaecology, Radiotherapy and Medicine, Queen Elizabeth Medical Centre, Birmingham, UK.*

Patients with inoperable epithelial ovarian cancer (EOC) have an inferior prognosis compared with those whose disease is optimally debulked at primary surgery. Recent WMOCG trials have shown that chemotherapy (CT) can render inoperable disease operable, but that secondary surgery carried out 6+ months after primary laparotomy was not associated with improved survival. Early second surgery (Intervention Debulking Surgery – IDS) carried out as soon as CT has produced sufficient cytoreduction to make this feasible may be a more logical approach since the emergence of a clone of tumour cells resistant to further CT may not have occurred. Thirty-seven patients with residual ovarian cancer following primary surgery received three courses *cis*-platinum 75 mg m$^{-2}$, adriamycin 50 mg m$^{-2}$ and bleomycin 15 mg m$^{-2}$ (PAB), given by intravenous infusion with hydration every three weeks. This was followed by six courses of escalating cyclophosphamide (1 g m$^{-2}$ in 0.5 g m$^{-2}$ increments) (esc-C) given three-weekly. IDS was carried out in suitable patients as soon as the surgeon felt that further surgery was possible.

Thirty-six patients are currently evaluable for response and toxicity. Sixteen patients were not considered for IDS. Twelve patients had <2 cm peritoneal seedlings only following primary surgery, and 4 patients had macroscopic disease (3 intrahepatic, 1 para-aortic nodes), which was felt to be unresectable. These received the chemotherapy programme only. Twenty patients who had undergone 'biopsy only' at primary laparotomy were considered for IDS after the second or third course of PAB if the referring gynaecologist felt that sufficient cytoreduction had occurred to make surgery feasible. Thirteen had 'good' surgical results at IDS, 10 no macroscopic disease, and 3 <2 cm macroscopic disease following IDS. One patient had inoperable disease, and IDS was not performed in 6 patients because of progression (3), or medical condition precluding surgery (3). Overall 25/36 (70%) patients (12 at primary laparotomy, 13 following IDS) had disease <2 cm or no macroscopic disease within 12–14 weeks of diagnosis, at a time when further effective CT could then be given. Toxicity was tolerable and predictable. Five patients experienced WHO grade 3 nausea and vomiting. Alopecia was reversible in all cases. WHO grade 3 myelosuppression occurred on 8 occasions in 6 patients. Ten patients had infective episodes which responded to antibiotics. Six patients had greater than 30% reduction in creatinine clearance. One patient had a pulmonary embolus following IDS. There were no treatment related deaths. Although follow up is short, no patient has so far progressed in the IDS group, and the encouraging initial results have led us to initiate a randomised trial to test this treatment programme.

**The prognosis of untreated stage I ovarian epithelial cancer**

C.J. Gallagher, M. Burnell, E. Wiltshaw
& J. Staffurth

*Gynaecological Unit, Royal Marsden Hospital, Fulham Road, London SW3 6JJ, UK.*

The value of adjuvant therapy for stage I ovarian epithelial carcinoma following optimal surgery and clinical investigation is unproven. A policy of no post-surgical therapy for all stage I epithelial carcinomas was adopted in this department in 1979. The aim was to follow newly diagnosed FIGO stage I patients with laparoscopies every 6 months for 2 years; to determine their survival, rate of relapse, salvage rate and the morbidity/mortality of repeated laparoscopies. This group comprises 33 patients with stage Ia–Ic, in whom surgical staging included a TAH and BSO in 32 with a negative post-operative ultrasound or lymphangiogram in 29. Borderline tumours were excluded. Only 7 patients had well differentiated stage Iai disease. A median of 3 laparoscopies per patient were performed. Bowel perforation requiring laparotomy occurred in one. Seven have relapsed with a median follow-up of 39 months. Five out of the 7b relapses were detected by laparoscopy. The other 2 patients relapsed at 30 and 41 months. Relapse was not related to substage, histology or tumour differentiation. Six patients were treated at relapse with single agent chemotherapy – *cis*-platinum 100 mg m$^{-2}$ or carboplatin 400 mg m$^{-2}$. A complete remission was achieved in 3 patients who remain disease free for 12 to 41 months. Two patients have died from their disease. This represents a 2 year survival of 94% and disease free survival of 79%. Post-operative adjuvant treatment has been avoided in these patients with stage I ovarian cancer. Early detection of relapse at laparoscopy has enabled the successful salvage of 3/6 patients with chemotherapy.

**Chemotherapy with *cis*-platinum, vincristine, methotrexate and bleomycin (POMB) for advanced carcinoma of the cervix**

G.J.S. Rustin[1,2], E.S. Newlands[2] & Barbara Southcott[2]

[2]*Departments of Medical Oncology and Radiotherapy, Charing Cross Hospital, London and* [1]*Regional Radiotherapy Centre, Mount Vernon Hospital, Northwood, Middlesex, UK.*

Because of the poor results of surgery ± radiotherapy in patients with squamous cell carcinoma of the cervix who have lymph node involvement or recurrent disease the additional use of chemotherapy has been investigated. Twenty-three patients were treated up to October, 1985, of whom 14 had received prior radiotherapy (recurrent). They received 2–6 courses of vincristine 1.0 mg m$^{-2}$ and methotrexate 300 mg m$^{-2}$ as 12 h infusion on day 1, followed by folinic acid rescue, bleomycin 30 mg as 48 h infusion days 2, 3 and *cis*-platinum 100 mg m$^{-2}$ as 12 h infusion on day 4. One, who had WHO performance of 3, died one month after the start of reduced dose chemotherapy and is not assessable for response. Of the remaining 22 patients, 6 had a complete remission (IB, IIIB, 2 IVs and 2 recurrent IIIBs), 11 had a partial remission (1B, IV and recurrent IIB, IIIB and 7 IVs) and in 5 patients there was no change (2 IVs and recurrent IIIB and 2 IVs) for an overall objective response rate of 77%. Although 14 patients have since died of their disease, 3 patients with recurrent or stage IV disease are alive with no evidence of diease 3+ years off treatment. This has prompted us to further study the role of multiple modality therapy using chemotherapy as the initial treatment in patients with visceral or nodal involvement from carcinoma of the cervix.

**Improved response and survival using ifosfamide in the treatment of metastatic soft tissue sarcoma**

W.P. Steward[1], D.P. Deakin[2], R. Swindell[3], D. Crowther[1] & M. Harris[4]

[1]*CRC Department of Medical Oncology, Departments of* [2]*Radiotherapy,* [3]*Medical Statistics and* [4]*Histopathology, Christie Hospital & Holt Radium Institute, Manchester M20 9BX, UK.*

Over a 9 year period 55 patients (pts) with metastatic soft tissue sarcomas (STS) have been treated with a 4-weekly cyclophosphamide, adriamycin, vincristine and DTIC (CYVADIC) and 47 pts have been managed with single agent ifosfamide or with combinations of chemotherapy containing ifosfamide (Ifos).

Subjective toxicity for all Ifos combinations was significantly less than for CYVADIC and the duration of inpatients' stay was also reduced.

The response rate to CYVADIC was 37% and to Ifos-containing regimens was 50%. The median survival from starting CYVADIC was 7 months and from commencing Ifos was 12 months ($P = 0.04$). The median relapse-free survival for CYVADIC was 8 months and for Ifos was 12 months ($P = 0.2$).

Ifos is an important agent in the management of metastatic STS and should be considered for inclusion in all combination regimens.

**The HTLV-III receptor, mechanisms of infectivity, and possible modes of treatment**

A.G. Dalgleish[1], R.A. Weiss[1], P. Clapham[1], M. Marsh[1], P.J. Maddon[2], R. Axel[2]

[1]*Institute of Cancer Research, Royal Cancer Hospital, Chester Beatty Laboratories, Fulham Road, London SW3 6JB, UK and* [2]*Columbia College of Physicians and Surgeons, NYC, USA.*

Previous studies by our group have shown that the T4 antigen of the helper-inducer subset of cells is an essential component of the HTLV-III receptor (*Nature*, **312**, 763, 1984). Using cell lines transfected with the human T4 genome (*Cell*, **40**, 237, 1985), we have been able to show that mice fibroblasts and lymphocytes expressing the human T4 antigen are resistant to infection with HTLV-III. We have since obtained a variety of human cells which do not normally express T4 but which, following transfection with the T4 genome, now do so, Preliminary data suggest that T4 binds to the envelope gene of HTLV-III and that a further component not expressed on mouse cells may be required for infection.

The virus probably follows the endocytic pathway described for other retroviruses and studies using endocytic inhibitors that support these data will be presented. These agents are in distinct contrast to those that inhibit the reverse transcriptase of the virus and have been tested *in vitro* (and one *in vivo*) by our laboratory. Studies on how the virus evades effective immunologic responses previously reported (*Nature*, **316**, 69, 1985) will be updated with particular reference to the implications for vaccine development.

The mechanism(s) whereby the virus causes 'cancer' in 40% of AIDS patients will be discussed with special emphasis on the importance of the above studies to the development of Kaposi's sarcoma.

**Detection of malignant lymphoma in the liver: Correlation between magnetic resonance imaging at 0.08 Tesla and open liver biopsy**

M.A. Richards[1], J.A.W. Webb[2], R.H. Rezneck[2], S.E. Jewell[2], T.A. Lister[1] & P.F.M. Wrigley[1]

[1]*ICRF Department of Medical Oncology and* [2]*Department of Radiology, St Bartholomew's Hospital, London EC1A, UK.*

The assessment of liver involvement in patients (pts) with malignant lymphoma by clinical examination, conventional imaging techniques and biochemical tests of liver function is notoriously inaccurate. High false negative rates with percutaneous liver biopsy have been demonstrated. An accurate non-invasive method of detection of hepatic lymphoma would be of considerable benefit.

This study correlates the results of spin lattice relaxation time (T1) measurements made by magnetic resonance imaging (MRI) at 0.08 Tesla with the pathological findings from liver biopsy in pts undergoing laparotomy for malignant lymphoma.

Seventeen pts – 13 with Hodgkin's disease (HD) and 4 with non-Hodgkin's lymphoma (NHL) – and 24 healthy volunteers have been scanned to date. Six of the 17 pts had histologically confirmed hepatic lymphoma (2 HD, 4 NHL). All 6 had diffuse elevation of liver T1 (>3 standard deviations above the mean for healthy volunteers: $P<0.0001$). One of these pts had additional focal liver abnormalities.

Eleven pts had no evidence of lymphoma on wedge liver biopsy. Nine of these had liver T1 measurements within the normal range. The tenth pt had slight elevation of liver T1 and liver histology was compatible with chronic active hepatitis. The final pt had a normal liver biopsy, but marked elevation of liver T1, the significance of which is unknown.

These early results suggest that MRI at low field strength may be helpful in the detection of hepatic involvement by lymphoma.

**Intensive 6 week remission induction followed by 3-weekly consolidation therapy for high grade non-Hodgkin's lymphoma**

N.S. Stuart, G.R.P. Blackledge, J.A. Child, A. Simmons, S. Cartwright, D. Barnard, M. Cullen & J. Fletcher

*West Midlands and Yorkshire Lymphoma Group*

In an attempt to improve complete remission rate and overall survival we have used an intensive, 6-week, 4-drug remission induction regime (VAMP: vincristine 2 mg i.v. wks 1–6, adriamycin 50 mg m$^{-2}$ i.v. wks 1, 3, 5, methotrexate 250 mg m$^{-2}$, i.v. wks 1, 3, 5 with folinic acid orally for 3 days, prednisolone 60 mg/day wks 1–6 reducing over 10 days) followed by a 3-weekly non-cross resistant consolidation regime (CViVp: cyclophosphamide 1 g m$^{-2}$, vindesine 3 mg m$^{-2}$, etoposide 125 mg m$^{-2}$ all i.v. day 1, etoposide 250 mg m$^{-2}$ days 2, 3 – all repeated day 21). Patients received at least 3 courses of CViVp following CR. One hundred and twenty patients with high-grade (Kiel) non-Hodgkin's lymphoma have been treated with this regime. Mean follow-up is 21 months, all patients have completed treatment. Mean age of the group was 54 years (range 14–78). Twenty-five patients were stage (S) I/II all with bulky, symptomatic or abdominal disease, 89 were S III or S IV. Sixty-five had B symptoms.

At the end of VAMP 53 patients (46%) were in CR. Of the 50 in PR at the end of VAMP 20 achieved CR during CViVp. Overall best status achieved was CR 75 (65%), PR 33 (29%), fail 9 (7%). Median survival of responders and median relapse free survival have not yet been reached. The proportion of patients receiving greater than 75% of protocol doses for each drug was as follows; adr, 73%; vincr, 61%; meth, 64%; pred, 92%; vind, 70%; cyclo, 77%; VP-16, 73%. Toxicity was acceptable in both phases of treatment. No neutropaenia below $0.5 \times 10^9 l^{-1}$ was recorded in 58% of patients during VAMP; 72% during CViVp. No platelet count below $50 \times 10^{12} l^{-1}$ was recorded in 93% during VAMP; 95% during CViVp. Moderate or severe neuropathy occured in 25% of patients during VAMP. Septicaemic episodes occurred in 24/122 (19%) patients. Fourteen deaths occurred to which treatment contributed, in 8 of these other causes were also involved.

This regime appears effective and well tolerated. Results are as good as those achieved with any other regime particularly with regard to the large proportion of elderly patients and those with advanced stage disease.

**Etoposide containing combination chemotherapy (CT) for Hodgkin's disease (HD)**

T.J. Perren, P.J. Selby, E.K. Mbidde & T.J. McElwain

*Section of Medicine, Institute of Cancer Research, Royal Marsden Hospital, Surrey, UK.*

Thirty-nine patients (pts) (20 clinical stage (CS) IV, 9 CS III, 10 CS II – poor prognostic factors) were treated with OPEC (vincristine 1.4 $mg\,m^{-2}$ day (D) 1 and 8, prednisolone 40 mg/day D 1–14, etoposide 200 $mg\,m^{-2}$ orally (p.o.) daily D 1–5, chlorambucil 6 $mg\,m^{-2}$ p.o. daily D 1–14). In 30 (28 previously untreated pts and 2 relapsing 9 years after previous treatment) this was alternated with ChIVPP (an established CT regimen – O/C) while 9 pts relapsing after previous treatment received OPEC alone (O). Median follow up is 40 months; complete remission (CR) rate 20/28 (71%) of O/C pts and 5/9 O pts; median relapse free survival has not been reached for O/C and was 44 months for O; actuarial 3 year survival was 77% for O/C and 60% for O.

Twenty-nine patients relapsing from previous CT have been treated with HOPE-Bleo (adriamycin 40 $mg\,m^{-2}$, vincristine 1.4 $mg\,m^{-2}$ day 1 and 8, prednisolone 40 $mg\,m^{-2}$ daily D 1–8, etoposide 200 $mg\,m^{-2}$ p.o. daily D 1–4, bleomycin 10 $mg\,m^{-2}$ day 1 and 8). Median follow up 12 months; CR 14/29 (48%); median relapse free survival 11 months and 2 year actuarial survival was 54%.

Nausea and vomiting, alopecia and myelosuppression sufficient to delay treatment were seen in 49%, 100% and 18% of OPEC pts and 55%, 100% and 21% of HOPE-Bleo pts respectively.

Etoposide can be combined safely and effectively with other agents in both first line and relapse regimens in HD.

**Studies of high dose chemotherapy in multiple myeloma (MM)**

T.J. Perren[1], P.J. Selby[1], R.K. Mbidde[1], J.S. Malpas[2] & T.J. McElwain[1]

[1]*Department of Medicine, Institute of Cancer Research and Royal Marsden Hospital, Downs Road, Sutton, Surrey and* [2]*Department of Medical Oncology, St Bartholomew's Hospital, London EC1, UK.*

Forty-one previously untreated patients (pts) aged <63 years with MM were treated with HDM 140 $mg\,m^{-2}$ i.v. Median follow up is 16 months (R 4–47). Eleven (27%) achieved complete remission (CR – normal bone marrow morphology and unmeasurable paraprotein), and 21 (51%) partial remission (PR – >50% reduction in paraprotein and improvement in all other clinical features). CRs seem durable with no relapses to date whereas 10/21 PR patients have relapsed ($P<0.05$). All patients had nausea, vomiting, diarrhoea and mucositis as well as WHO grade 4 myelosuppression. Median recovery of leucocytes ($>1\times10^9\,l^{-1}$) and platelets ($>25\times10^9\,l^{-1}$) occurred at 28 and 24 days respectively. There were 8 treatment related deaths, usually from sepsis and/or bleeding. In a group of 15 heavily pretreated pts, the response rate was also high (66%) but there have been no durable remissions. In relapsed heavily pretreated pts we have used high dose methylprednisolone (HDMP – 1 $g\,m^{-2}$/i.v. daily ×5 repeated q. 21 days). Of 9 patients evaluable for response there were 4 PRs of short duration (2–6 months) with minimal toxicity. Our present study combines HDM with a single 5 day course of HDMP. Median follow up is 4 months; 9/12 pts are evaluable for response, 4 achieved CR and 5 PR. Overall there was no increased toxicity and in particular no treatment related deaths.

HDM with or without HDMP seems a promising treatment for young previously untreated pts with MM and may be superior to standard therapy.

**Measurement of psychological morbidity in patients with advanced cancer of the breast**

P. Hopwood & A. Howell

*Department of Psychiatry, University Hospital of South Manchester and CRC Department of Medical Oncology, Christie Hospital, Manchester, UK.*

The aim of the study was the evaluation of 2 screening instruments in the detection of psychological morbidity in patients with advanced breast cancer. The self-rating scales used were the Hospital Anxiety and Depression Scale (HADS. Zigmond & Snaith, *Acta Psychr. Scand.*, **67**, 361, 1983) and the Rotterdam Symptom Checklist (RCL. de Haes *et al.*, *Ned Tijd. Phsychol.*, **38**, 403, 1983).

Two hundred and ten out of two hundred and thirty-five patients completed both questionnaires on one or more occasions, an overall compliance of 89%. Both instruments identified 31–34% patients with high scores on at least one occasion, and a similar proportion of patients with borderline scores.

Eight-one patients were also assessed using a standardised psychiatric interview, the Clinical

Interview Schedule (CIS, Goldberg *et al., Br. J. Soc. Prev. Med.*, **24,** 18, 1970) to assess the validity of the two instruments. Twenty patients received a psychiatric diagnosis (depression or anxiety), 11 were borderline cases and 50 were psychologically well. The sensitivity of the RCL in identifying cases of morbidity was 75% with 80% specificity. For the HADS the sensitivity was 75% for depression and anxiety and the specificities 75% and 90% respectively. Both instruments performed less well in identifying borderline cases (60–73%).

We conclude that both instruments are equally accurate in detecting cases of psychological morbidity but identify slightly different patient groups. The HADS has a potential advantage in being able to discriminate cases of depression and anxiety. Both instruments require further refinement to be of use in detecting borderline cases.

### Quality of life after treatment for testicular cancer

N.S.A. Stuart, R. Grundy, C. Woodroffe & M.H. Cullen

*Queen Elizabeth Hospital, Edgbaston, Birmingham, B15 2TH, UK.*

Recent advances in chemotherapy have led to the prospect of cure for the majority of patients with non-seminomatous germ cell tumours. The immediate subjective toxicity of such chemotherapy is well known, but the long-term effects on quality of life have not been reported. By means of a category-rating type questionnaire we have assessed the effects of treatment for testicular cancer on 2 groups of patients. The study group comprised 36 patients (28 replies) who had received chemotherapy, the control group comprised 54 patients (34 replies) matched for age, social group and time since start of treatment, who had been treated with radiotherapy. Median time since start of treatment was 32 months in each group.

Results showed there to be no difference between the 2 groups in their assessment of their general health and fitness or in their job prospects since treatment. The majority of patients in both groups felt as fit and as healthy as before their illness and remained in full-time employment. There was no difference in the groups' assessment of changes in the quality of their relationships with wife, friends or family. Both groups reported an improvement in such relationships since treatment. Both groups reported that they worried more about the future in general and about their health since treatment, and felt they were more often depressed – all but the former being more marked in the control group. Hearing difficulties since treatment were more frequent in the study group (39%; controls = 12%; $P=0.05$) and 3 study patients reported Raynaud's phenomenon (controls = 0). At least 39% of the study group were still fertile, 7 having fathered children.

We are encouraged that current chemotherapy does not substantially increase the long-term emotional, psychological and social effects of diagnosis and treatment for testicular cancer. However, there is scope for reducing the physical side-effects of treatment.

### Psychiatric morbidity following mastecomy, the value of counselling

P. Whitford, J.M. McArdle & C.S. McArdle

*University Department of Surgery, Royal Infirmary, Glasgow, UK.*

Anxiety and depression are common following mastectomy. The incidence is higher in patients with a past history of depression, severe marital problems, social problems or who lack a close confidante. In this study two groups of patients, one of which received informal counselling from a Nursing Sister following mastectomy, were studied. Psychiatric morbidity was measured by means of self administered questionnaires, the general health questionnaire (GHQ) and the Leeds Scales of depression (LSD) and anxiety (LSA). Twelve months after mastectomy, psychiatric morbidity was present in 46% of control patients, depression in 40% and anxiety in 54% (Table). The incidence of depression and anxiety in patients receiving informal counselling was significantly lower. The incidence of risk factors was similar in both groups.

Psychiatric morbidity one year after mastectomy (%)

| | *GHQ* | *LSD* | *LSA* |
|---|---|---|---|
| Control ($n=35$) | 46 | 40 | 54 |
| Counselled ($n=42$) | 14[a] | 11[a] | 30 |

[a] $P<0.05$.

These findings suggest that the high incidence of anxiety and depression following mastectomy can be reduced by informal counselling.

**Transcatheter embolisation to control severe bleeding in breast cancer**

E.M. Rankin[1], R.D. Rubens[1] & J.F. Reidy[2]

[1]*Department of Clinical Oncology and* [2]*Department of Radiology, Guy's Hospital, SE1 9RT, UK.*

Haemorrhage from fungating breast cancer is uncommon but alarming. Recourse to ligation of the internal mammary artery when simple measures fail (e.g. pressure bandages, suture of bleeding point) may be inappropriate in frail patients with advanced disease.

Transcatheter embolisation of the arteries supplying the tumour offers an alternative approach to the problem. The internal mammary artery is approached via the ipsilateral femoral artery using interventional radiology techniques and the tumour blood supply is demonstrated. Various agents including gelfoam, steel coils, Ivalon, alcohol and dextrose may be injected to produce occlusion and sclerosis. Since only a local anaesthetic with systemic sedation and analgesia are needed the problem of general anaesthesia is avoided.

Eight patients (ages 44–74 have been treated; in 7 immediate control of bleeding was secured, and in 6 no further haemorrhage occurred (longest follow up to date 22 weeks). In 1 patient another haemorrhage 2 months later was stopped by embolisation of the newly developed collateral circulation to the tumour. The tortuous vascular anatomy in 1 patient prevented entry into the internal mammary artery and so embolisation was not possible. The only side effect of the procedure has been some pain following injection of sclerosant. Subsequent response to chemotherapy is not affected by embolisation. This is a safe procedure and prophylactic embolisation should be considered in patients at risk.

**Single high dose aminohydroxypropylidene diphosphonate (APD) infusions to treat cancer-associated hypercalcaemia**

B.M.J. Cantwell & A.L. Harris

*University Department of Clinical Oncology, Regional Radiotheapy Centre, Newcastle General Hospital, Newcastle upon Tyne, UK.*

Ralston *et al.* (*Lancet*, **ii**, 907–910, 1985) reported intravenous (i.v.) daily infusions of APD to treat cancer-associated hypercalcaemia, and serum calcium levels fell significantly by day 2. Side effects of daily i.v. infusions of APD include transient fevers and drip site thrombophlebitis.

We have treated more than 15 patients with cancer-associated hypercalcaemia. Analysis of first 10 patients treated is as follows: mean age 54.2 years, range 41–76, comprising 3 women with breast cancer and 7 men. APD 30 mgs in 500 mls of 0.9% saline was infused i.v. over 2 h. Normocalcaemia (serum calcium 2.25–2.75 $mmol\,l^{-1}$) was achieved with APD in all but 2 patients who, despite substantial reductions in serum calcium levels did not achieve normocalcaemia. Mean (SEM) for pre-APD and post-APD serum calcium levels were 3.16 (0.11) and 2.56 (0.10) $mmol\,l^{-1}$ respectively, and for maximum pre-APD and minimum post-APD levels 3.29 (0.12) and 2.38 (0.10) $mmol\,l^{-1}$ respectively. Significant fall in serum calcium levels occurred with APD $P = <0.005$ for both pair groups (Wilcoxon tests, 2-tailed probabilities). Time to normal or minimum serum calcium was 1–8 days from start of APD therapy, and in those patients achieving normocalcaemia serum calcium remained normal for >5 weeks in 3 patients. There were no side effects with APD except for mild transient fever within 1 day of administration in 3 patients. Single i.v. infusions of 30 mg APD every 2–3 weeks may be adequate to treat mild to moderate cancer-associated hypercalcaemia. This would be more convenient for patients than daily treatments and would lessen the risk of drip site thrombophlebitis.

**Weekly oral idarubicin in advanced breast cancer**

D.B. Smith[1], A. Howell[1] & G. Thomas[2]

[1]*CRC Department of Medical Oncology, Christie Hospital, Manchester and* [2]*Farmitalia Ltd, UK.*

Idarubicin is an anthracycline analogue with approximately 30% bio-availability when administered orally. The major metabolite of Idarubicin, 13-OH 4DMDNR, is of equal anti-tumour activity to the parent compound in animal systems and since it is still present in the serum 7 days after dosing it was thought that a weekly schedule might be of benefit by mimicking a continuous infusion of cytotoxic activity. Thirty-eight patients with advanced breast cancer who had not received prior chemotherapy were treated with Idarubicin 15 $mg\,m^{-2}\,wk^{-1}$. Median age was 61 (33–79) and dominant sites of disease were: Stage IV primary 9, bone 9, lung 6, cutaneous 6, lymph nodes 3 and retroperitoneal infiltration 2. Fourteen patients had previously responded to hormone therapy while 18 had shown no response and 6 had had no hormone treatment. The major toxicity was nausea ± vomiting affecting 32% of courses, 4 patients (10.5%) requiring dose reduction for intractable symptoms. Three patients noted partial alopecia and there were no episodes of cardiac failure. Neutropenia (bc $2.5 < 10^9 l^{-1}$) resulted in 9 dose reductions and 9 treatment delays. There was no platelet toxicity and no episodes of infection. With a median follow-up of 28 weeks (8–60) there has been 1 CR (duration 11+ months) and 5 PRs (duration 11+, 7+, 6+, 3+ and 3+ months), an overall response rate of 15%. However, a further 6 patients had no change for 6+ months and thus 30% either responded or had static disease. Oral Idarubicin in a weekly schedule is comparatively non-toxic and has moderate activity in advanced breast cancer but does not appear to be superior to a q. 21 day regimen.

**Phase II trial of mytomycin C (MC) and 5-fluorouracil (5-FU) in advanced previously treated breast cancer**

E.J. Bayliss, M.E. Kerr, J.F. Smyth & R.C.F. Leonard

*University Department of Clinical Oncology, Western General Hospital, Edinburgh, UK.*

Patients (pts) with advanced breast cancer and who relapsed after or became resistant to adriamycin regimes have been treated with MC/5-FU (MC, $6 mg m^{-2}$ max 10 mg q. 6/52 and 5-FU $1 gm m^{-2}$ q. 3/52). Twenty of 25 pts have received minimum one full course, 19 evaluable for response. Five patients received course 1A only at half dose and died of disease (4 pts) or GI haemorrhage (1 pt at day 5). Fourteen of 19 pts had visceral (10 pts) and/or multiple sites involved. Patients received 1–5 cycles (median 2) producing 6/19 PR and 13/19 NR. The median duration of response is 84 days (34–158 days) and survival median 131 days (74–486 days). Both responders and non-responders had similar extent of prior treatment (PR median 8, range 6–12 cycles; NR median 6, range 3–21 cycles). Toxicity was acceptable for the 19 evaluable pts; nausea and vomiting, grades 0/I 68%, II/III 32%; neutropenia grades 0/I 42%, II/III 52%, IV 6%; thrombocytopenia 0/I 81% II/III 19%. Alopecia and clinical pulmonary deterioration did not occur. In summary MC/5-FU is active (OR 32%) and has acceptable toxicity for patients who have been previously treated for advanced breast cancer.

**The effect of aminoglutethimide (AMG) on liver function tests (LFTs) in advanced breast cancer**

E.J. Bayliss[1], P. Levack[2], U. Chetty[2] & R.C.F. Leonard[1]

*Department of [1]Clinical Oncology and [2]Clinical Surgery, Western General Hospital, Edinburgh, UK.*

Abnormal LFTs occur in patients (pts) on AMG, being attributed variously to hypersensitivity, induction of liver enzymes, hepatic toxicity or unmasking of hepatic metastases (mets). We have reviewed our experience of AMG in breast cancer and 24 pts with no clinical and/or ultrasonic evidence of liver mets were considered evaluable with normal ($<40 u l^{-1}$) alanine amino transferase (ALT) and normal gamma glutamyl transpeptidase (GGT, $<35 u l^{-1}$) pre-treatment. All had either serial LFTs performed during the first 6 months of AMG or at least one measurement during AMG and 1 or more measurements after cessation of AMG. Three pts with known liver mets and normal LFTs were also studied. In all 24 pts GGT rose by minimum 75% on AMG from mean $18.5 u l^{-1}$ (range 5–35) pre-treatment to highest measured of mean $128 u l^{-1}$ (range 23–471). Corresponding values for ALT were $20.7 u l^{-1}$ (10–37) at start and $51.3 u l^{-1}$ (11–202) maximum (>50% rise in 13). For alkaline phosphatase the initial mean was $94 u l^{-1}$ (43–205) and maximum $130.6 u l^{-1}$ (41–1033) respectively and those with no bony mets $82 u l^{-1}$ (43–205) at start and 106 (41–286) maximum (>25% rise in 6, >10% rise in 14 patients). In 5 pts GGT was measured as rising at 2 weeks after start of treatment, 11 pts had sufficient data to show peak abnormalities between 1–3 months for all enzymes and thereafter either falling rapidly, especially when AMG stopped, or remaining abnormal up to 12 months in some of the pts who continued AMG. The 3 pts with known liver mets and normal LFTs showed similar patterns before developing clinically progressive disease in liver and symptoms thereof. The transience of elevation suggests enzyme induction rather than direct hepatotoxicity. The phenomenon can be misconstrued as progressive liver disease in this group of 'at risk' patients.

**Beneficial effect of high dose tamoxifen in patients with advanced breast cancer not responding to standard doses**

Sylvia M. Watkins

*Department of Medical Oncology, Lister Hospital, Stevenage, UK.*

The effect of increasing the dose of tamoxifen from 20–40 mg to 90 mg daily was studied in 25 post menopausal breast cancer patients (aged 46–83), whose disease was progressing on the lower ('standard') doses. On standard doses of tamoxifen there had been 3 complete, 3 partial remissions (PR), 9 with stable disease (SD) all followed by progressive disease (PD); the remaining 10 progressed relentlessely (UICC criteria). On the high dose there was 1 PR lasting 5 months and 16 SD lasting 3 to 19 months (median 6 months). Side effects were slight (lethargy, tiredness, loss of taste and hot flushes in 4 patients). There was no correlation between response to standard dose and high dose tamoxifen, and no correlation between response and plasma levels of tamoxifen.

In spite of the low objective response rate on the higher dose, two-thirds of the patients had a period of stable disease lasting sometimes many months, and in view of the minimal side effects this was a worthwhile outcome of treatment in this group of patients. Comparable results were found by Stewart *et al.* (*Cancer Treatment Rep.*, **66**, 1445, 1982).

Increasing the dose of tamoxifen is a useful and acceptable approach in cases of primary and secondary failure of tamoxifen treatment, and well worth trying before switching to other forms of endocrine therapy or chemotherapy, all of which have more serious side-effects.

**Very high dose cyclophosphamide (HDCY) followed by *cis*-platinum (CP) in advanced ovarian cancer**

R.J. Osborne[1], B.D. Evans[2], C.D. Wood[1], C.J. Gallagher[2], M.L. Slevin[1], J.H. Shepherd[3] & E. Wiltshaw[2]

*Departments of [1]Medical Oncology and [3]Gynaecological Oncology, St Bartholomew's and Hackney Hospitals and [2]Department of Medicine, Royal Marsden Hospital, London, UK.*

Twenty patients (pts) with previously untreated ovarian cancer (12 stage III, 8 stage IV) received intensive chemotherapy after initial surgery (small residual 11, bulk residual 9). *Treatment Plan* HDCy $7\,g\,m^{-2}$ with mesna $10.5\,g\,m^{-2}$ for 2 courses, followed by re-evaluation with second look laparotomy followed by CP $100\,mg\,m^{-2}$ for 5 courses. Two initial pts received 3 cycles of HDCy, 15 pts received 2 cycles of HDCy, 3 pts received 1 cycle HDCy. Fifteen of 20 pts subsequently received CP (mean 4 courses). *Results HDCy* Pathologically documented complete remission (PDCR) – 3/14 pts (21%). Partial remission (PR) 8/14 (58%). Progression 3/14 (21%). Unassessable – (no second look laparotomy) 6 pts. *Toxicity* There was evidence of cumulative marrow toxicity.

| *Course HDCy* | *Pts* | *Days neutrophils* $1\times10^9\,l^{-1}$ *mean (range)* | *Days platelets* $1\times10^9\,l^{-1}$ *mean (range)* |
|---|---|---|---|
| 1 | 20 | 12.2 (8–16) | 8.0 (0–13) |
| 2 | 15 | 19.6 (8–115) | 25.8 (3–116) |
| 3 | 2 | 20 (14–26) | 59.5 (19–100) |

Two pts died from treatment related causes (1 septicaemia, 1 persistent bone marrow hypoplasia). *Overall Results After HDCy and CP* (20 pts). PDCR 4 pts. Clinical CR 4 pts. Alive in relapse 4 pts. Died 7 pts. *Survival* Median survival of whole group 20 months. *Conclusion* HDCy is toxic therapy, and the pathologically documented complete remission rate is not greater than that expected with conventional therapy.

**A pilot study of carboplatin (JM8) and chlorambucil (CLB) in epithelial ovarian carcinoma**

S.B. Kaye, M. Harding, S.J. Harland, J. Graham, Lesley Mill, A. McLean, R. Kennedy, I. Duncan, J. Kennedy & M. Soukop

*Departments of Medical Oncology and Gynaecology, University of Glasgow, Glasgow Royal Infimary and Ninewells Hospital, Dundee, UK.*

To date, 32 patients with previously untreated ovarian carcinoma (FIGO Stages II, III or IV) have received JM8 $300\,mg\,m^{-2}$ and chlorambucil 10 mg daily for 7, 10 or 14 days to assess the toxicity of this combination prior to a randomised Phase III trial comparing the efficacy against that of single agent JM8. The major toxicity was myelosuppression and chemotherapy was delayed beyond the planned 4 weekly interval by WHO Grade I leukopenia. Subsequent courses were given with modified chlorambucil dosage (50 or 70 mg).

| *JM8 + CLB* | *No. of patients* | *Total treatment* | *No. cycles* | *Mean nadir delayed (dose reduced)* | *WBC* | *Plats* |
|---|---|---|---|---|---|---|
| 7 days | 16 | 9 | 49 | 6 (7) | 3.2 | 131 |
| 10 days | 5 | 1 | 29 | 16 (12) | 3.1 | 135 |
| 14 days | 11 | 7 | 36 | 13 (16) | 3.1 | 154 |

Treatment was well tolerated on an outpatient basis in most cases with WHO Grade 0–2 nausea and vomiting, a single patient experienced moderate alopecia. A clinical response has been documented in 10 of 16 evaluable patients. The study is ongoing to confirm that the combination of JM8 300 mg m$^{-2}$ with chlorambucil 10 mg daily for 7 days is as feasible as these preliminary data suggest.

### A randomised comparison of three platinum analogues in combination with cyclophosphamide in patients with advanced epithelial ovarian cancer

H. Anderson[1], J. Wagstaff[1], D. Crowther[1] & M. Timms[2]

[1]*CRC Department of Medical Oncology, Christie Hospital, Manchester, M20 9BX and* [2]*ENT Department, Manchester Royal Infirmary, Oxford Road, Manchester 13, UK.*

Twenty patients have now been entered into each of the three arms of a pilot study comparing *cis*-platinum 100 mg m$^{-2}$ with CHIP 240 mg m$^{-2}$ and CBDCA 300 mg m$^{-2}$ in combination with cyclophosphamide 600 mg m$^{-2}$. Cycles of therapy are repeated at four weekly intervals for a total of six courses. This abstract (Nov., 1985), is of data from 42 patients who have completed therapy, and the data will be updated by March, when almost 60 patients will have completed therapy.

| | Cis-*plat* | *CBDCA* | *CHIP* |
|---|---|---|---|
| Total number of patients | 14 | 11 | 17 |
| No. patients | | | |
| dose reduction/delay | 6 | 6 | 15 |
| Response | | | |
| CR/PR | 11 | 7 | 11 |
| Stable | 0 | 2 | 0 |
| Progression | 1 | 0 | 5 |
| Not evaluable | 2 | 2 | 1 |
| Toxicity | | | |
| Nausea & vomiting | 14 (3)[a] | 11 (2) | 17 (2) |
| Alopecia | 14 (3) | 10 (2) | 15 (2) |
| Diarrhoea | 2 | 1 | 13 (2) |
| Tinnitus | 12 | 5 | 3 |
| Deafness | 6 | 2 | 0 |
| Paraesthesiae | 9 | 1 | 3 |

[a]Number of patients followed by median WHO toxicity grade in brackets. Haematological toxicity has been seen in all arms and is most severe in CHIP patients necessitating frequent dosage reduction in therapy and in CBDCA patients to a lesser extent. Renal toxicity has only been seen in *cis*-platinum treated patients.

### Successful management of metastatic and primary germ cell tumours in the brain

G.J.S. Rustin, E.S. Newlands, K.D. Bagshawe, R.H.J. Begent & S.M. Crawford

*Cancer Research Campaign Laboratories, Department of Medical Oncology, Charing Cross Hospital, London, UK.*

Nine men and one woman with brain metastases from previously untreated non-seminomatous germ cell tumours have been treated between 1977 and 1984. All the men had lung metastases. Nine patients had elevated serum values of human chorionic gonadotrophin (HCG), the level was greater than 40,000 iu l$^{-1}$ in seven. They were treated with sequential combination chemotherapy, either POMB/ACE (Newlands *et al.*, *Lancet*, **i**, 948, 1983) or EP/OMB (etoposide 200 mg m$^{-2}$, *cis*-platinum 100 mg m$^{-2}$ alternating every 8–10 days with vincristine 1 mg m$^{-2}$ methotrexate and bleomycin 300 mg as 48 h infusion) but no radiotherapy. The methotrexate was given at a dose of 1 g m$^{-2}$ with folinic acid rescue starting at 32 h and intrathecal methotrexate was given during courses not containing intravenous methotrexate. Eight of 10 patients are alive, off treatment with no evidence of active disease of whom 5 have been in remission and off treatment for more than 2 years.

Two patients with primary intracranial non-seminamatous germ cell tumours were treated in a similar fashion. One patient died from enlargement of differentiated teratoma, the other is alive 15+ months off treatment with no evidence of disease.

These results, which are better than any previously reported, indicate that chemotherapy is the preferred treatment of primary or metastatic non-seminomatous germ cell tumours of the brain and that only rarely will these patients benefit from surgery or radiotherapy.

### The effect of dose on the bioavailability of oral etoposide

M.L. Slevin, S.P. Joel, M. Richards, V.J. Harvey, R. Whomsley & P.F.M. Wrigley

*ICRF Department of Medical Oncology, St Bartholomew's and Hackney Hospitals, London, UK.*

Previous studies from this department have suggested that the bioavailability of oral etoposide may decrease as the administered dose increases (Harvey *et al.*, *ASCO*, **3**, 24, 1984, *Cancer Chemother. Pharmacol.*, in press). A study to further investigate this observation and to determine the dose at which the bioavailability decreases has been conducted. Ten patients (pts) with malignant mesothelioma who were receiving single agent etoposide in a Phase II study have been investigated. Each pt was studied after oral etoposide at a total dose of 100, 200, 300, 400 and 600 mg. The order of administration of these doses was randomised. Etoposide was administered at 9 am after an overnight fast. Food and drink were allowed *ad libitum* 2 h after dose. Etoposide concentrations in plasma and urine were measured using an HPLC assay (Harvey *et al.*, *J. Chrom.*, **339**, 419, 1985).

| *Results* | *100 mg* | *200 mg* | *300 mg* | *400 mg* | *600 mg* |
|---|---|---|---|---|---|
| Mean AUC ($\mu g\, ml^{-1} \times h\, m^{-2}$) | 38.8 | 68.9 | 93.5 | 105.3 | 139.5 |
| % increase over 100 mg | — | 78% | 141% | 171% | 260% |
| Predicted increase over 100 mg | — | 100% | 200% | 300% | 500% |
| T test of observed *vs* expected AUC | — | $P=0.3$ | $P=0.06$ | $P<0.0001$ | $P<0.0001$ |

Peak etoposide concentrations and urinary concentrations followed a similar pattern and did not achieve the predicted levels. This study confirms the earlier observation that oral etoposide bioavailability decreases with increasing dose in most patients.

**Decreased half life of ifosfamide (I) after daily oral administration**

M. Lind[1], T. Cerny[1], J. Margison[2], N. Thatcher[1] & P.M. Wilkinson[2]

[1]*CRC Department of Medical Oncology and* [2]*Department of Clinical Pharmacology, Christie Hospital & Holt Radium Institute, Manchester M20 9BX, UK.*

Six patients with small cell lung cancer were treated with (I) 2 g p.o. daily for three days. Serial serum and urine samples were collected over the first 96 h after administration of the first dose and concentrations of (I) assayed by HPLC using a method developed in this laboratory. We have previously showed 100% bioavailability of (I) for doses up to 2 g and that the elimination phase was identical after equivalent oral or i.v. bolus doses. Therefore, total clearance, distribution volume and half-time can all be derived from the oral concentration/time profile. These values are given in the table. There was a progressive decrease in AUC by 40% of the initial value at day 3. This was associated with a decreased plasma half-time and increased clearance. The distribution volume remained unchanged. Similar observations have been shown for cyclophosphamide and we suggest that the kinetic changes are due to induction of (I) metabolism.

Pharmacokinetics of fractionated (I)

| | *Day 1* | *Day 2* | *Day 3* |
|---|---|---|---|
| T 1/2 h | 6.8 | 5.3 | 4.8 |
| AUC $\mu g\, l^{-1}\, h$ | 608 | 471 | 383 |
| $CL_{tot}$ $ml\, min^{-1}$ | 54 | 70 | 86 |
| $V_D L^1 s$ | 32 | 31 | 35 |

**An *in vitro* test for activity of agents potentially useful in treating human renal cell carcinoma**

D. Heinemann[1], M.A. Ferro[2], A.V. Kaisary[3], P.J.B. Smith[2] & M.O. Symes[1]

*Departments of* [1]*Surgery and* [2]*Urology, University of Bristol, Bristol Royal Infirmary, Bristol BS2 8HW and* [3]*Department of Urology, University of Oxford, UK.*

Tumour cell suspensions were prepared from 19 renal cell carcinomas by disaggregation with collagenase and DNase. Relatively pure suspensions of carcinoma cells were then separated following centrifugation of the mixed cell suspension on a Nycodenz (Nyegaard & Co. As, Oslo) column. Aliquots of $10^5$ tumour cells were cultured *in vitro* with graded concentrations of 5, therapeutic agents for 24 h. Thereafter the cells were washed free of the agent and resuspended in 2 ml of methionine - free MEM with 2 $\mu$Ci of $^{75}$Se (as Seleno-methionine, SCIP Amersham Int plc) for 48 h. Incorporated radioactivity in the pellet, as a measure of protein synthesis, was compared between cells exposed to a drug and control cells cultured in medium alone.

Lai *et al.* (*Proc 1st Conference on Neoadjuvant Chemotherapy*, Paris, 1986) using a mouse mammary carcinoma, showed that >70% inhibition of $^{75}$Se uptake predicted a significant anti-tumour action of the drug *in vivo*. We found depo-provera,

1.0 $\mu$g ml$^{-1}$ produced >70% inhibition of protein synthesis in 3/19 tumours. The corresponding proportion for vinblastine, 1.0 $\mu$g ml$^{-1}$, was 2/14 and for methotrexate 400 $\mu$g ml$^{-1}$, 1/13 tumours. However, adriamycin, 1.0 $\mu$g ml$^{-1}$, was effective against 4 of 6 tumours and the related drug, novantrone, against 4 of 8 at 1.0 $\mu$g ml$^{-1}$ and 6/9 for 10.0 $\mu$g ml$^{-1}$. The use of novantrone to treat metastatic renal cell carcinoma might thus be investigated.

**The role of reduced glutathione as a determinant of cellular sensitivity to 'activated' cyclophosphamide – A possible role for acrolein**

T.R. Crook[1], R.L. Souhami[2] & A.E.M. McLean[1]

[1]*Toxicology Laboratory, University College London, London WC1 and* [2]*Department of Radiotherapy and Oncology, University College Hospital, London WC1, UK.*

A dual culture system of rat hepatocytes and K562 human leukaemia cell line has been used to investigate the intracellular metabolism and cell cytotoxicity of cyclophosphamide (CP). After exposure to activated CP alkaline elution analysis of leukaemia cell DNA demonstrated substantial levels of DNA single-strand breaks, in addition to DNA interstrand cross-links and DNA-protein cross-links. Acrolein was shown to be the metabolite of CP which caused single-strand breaks. Acrolein was also a highly effective depletor of cellular reduced glutathione (GSH), but phosphoramide mustard had no effect on cellular GSH content. K562 cells depleted of GSH either by Buthionine sulfoximine (BS) or BCyNU show greatly increased sensitivity to the cytotoxic and DNA damaging effects of activated CP, but not to phosphoramide mustard. Similarly, the cytotoxic and DNA cross-linking effects of 'activated' CP, but not phosphoramide mustard, were antagonised by cysteine. These results suggest that GSH depletion either by exposure to agents such as BSO or BCyNU, or by intracellularly released acrolein itself, increases the conversion of 4-OH-CP into cytotoxic metabolities. These findings are of importance in devising novel strategies for overcoming resistance to CP.

**$^{111}$Indium labelled monoclonal antibody to placental alkaline phosphatase is of clinical value on the detection of neoplasms of testis, ovary and cervix**

Teresa R.B. Pawlikowska & A.A. Epenetos

*On behalf of the Hammersmith Oncology Group, Royal Postgraduate Medical School, Hammersmith Hospital and the Imperial Cancer Research Fund, London, UK.*

Indium-111 labelled monoclonal antibody H17E2 raised against placental alkaline phosphatase and testicular placental alkaline phosphatase has been used to image patients with carcinoma of the testis, ovary and cervix. Forty-one patients have been studied with radioimmunoscintigraphy. Imaging of neoplastic lesions was achieved in the majority of patients with active disease. In two patients with normal conventional radiology, positive monoclonal antibody scans were obtained which located sites of disease recurrence, confirmed by surgical lymphadenectomy. Conversely, in one patient who had a normal antibody scan together with abnormal conventional radiology, a congenital G.I. cyst was found at operation. Therefore, this method appears to be useful in the assessment of disease status in patients with PLAP-positive neoplasms.

**Antibody guided irradiation – Fact or fiction**

S. Stewart & A.A. Epenetos

*On behalf of the Hammersmith Oncology Group, Royal Postgraduate Medical School, Hammersmith Hospital and the Imperial Cancer Research Fund, London, UK.*

To maximise the therapeutic potential of monoclonal antibodies their administration into body cavities has been explored.

Patients with advanced carcinoma of the ovary were treated intraperitoneally with $^{131}$Iodine labelled monoclonal antibody given singly or as a mixture, depending on immunohistochemistry. Symptomatic benefit was observed in most cases. Four out of six patients with Stage III disease have achieved complete remission at two years following therapy. Toxicity was noted if more than 100 mCi of radioactivity was administered (i.e. reversible diarrhoea, leucopenia and thrombocytopenia).

Patients with pleural and/or pericardial effusions of diverse neoplastic aetiology have been treated with local instillation of monoclonal antibody. (20–100 mCi $^{131}$I). Ten out of thirteen patients achieved remission. This treatment appears to be effective in terms of alleviating fluid reaccumulation.

Monoclonal antibody (EGFR1) against epidermal growth factor receptor has been used to treat five patients with Grade III or IV gliomas. Three patients showed significant symptomatic improvement with this therapy.

These three areas are now being explored in prospective studies to determine the clinical value of monoclonal antibody therapy.

**Radioimmunotherapy of colorectal cancer**

R.H. Begent[1], J.A. Ledermann[1], K.D. Bagshawe[1], F. Searle[1], P.A. Keep[1], A.J. Green[1], R.G. Dale[2] & M.G. Glaser[3]

[1]*Department of Medical Oncology,* [2]*Department of Medical Physics and* [3]*Department of Radiotherapy, Charing Cross Hospital, London W6, UK.*

Antibodies directed against carcinoembryonic antigen (CEA) will localise in colorectal cancer when given intravenously. They can be labelled with $^{131}$Iodine ($^{131}$I) to deliver therapeutic doses of radiation. However, much of the radiolabelled antibody persists in normal tissues giving them an unacceptable dose. Imaging doses of radiolabelled (first) antibody to CEA can be cleared from normal tissues by giving a second antibody directed against the first. The purpose of this study was to investigate whether second antibody could be used to improve the therapeutic ratio of radioimmunotherapy with therapeutic doses of radiolabelled antibody. Five patients with unresectable colorectal cancer were given 2.5 mg of goat anti-CEA labelled with 40–55 mCi $^{131}$I. Twenty-four hours later 7.5 mg of horse anti-goat second antibody was given. Clearance of first antibody was accelerated by second antibody in 4/5 as shown by falling levels of radioactivity in the blood and by gamma camera imaging. Radioactivity was cleared in the urine and a smaller amount in the faeces. Radioactivity was retained in the tumours in all patients and in one, pain relief and reduction in serum tumour marker levels was noted. One patient had a transient fever and another a rigor. There was no other toxicity. This forms a basis for a dose escalation study to determine whether a useful antitumour effect can be achieved.

**Endometrial stromal sarcoma – Response to medroxyprogesterone after danazol treatment**

D. Parker

*Oncology Unit, Bradford Royal Infirmary, Bradford BD9 6JR, UK.*

Endometrial stromal sarcoma is an unusual tumour whose histological appearance may belie its aggressive behaviour.

A 37-year old patient is described who developed invasion of the vagina and bladder with bilateral hydronephrosis four years after hysterectomy. The histology of the uterus had shown 'stromal myosis'. Further histological specimens had shown no clear evidence of malignancy until sections of the bladder wall were examined after hydronephrosis had developed.

The tumour had progressed in the face of danazol 200 mg daily for 10 months and tamoxifen 20 mg bd for 2 months. Medroxyprogesterone 100 mg daily resulted in haematuria followed by gradual resolution of her symptoms. There was objective evidence of tumour regression on CT scan and some improvement in renal function.

This case illustrates the importance of positive diagnosis of endometrial stromal sarcoma. The contrast between progression on danazol and regression on progestogens is important in view of the use of danazol for endometriosis. Patients with rare but responsive tumours are a significant group.

**Phase II trial of carboplatin (JM8) in the treatment of patients with mesothelioma (M)**

E.K. Mbidde, I.E. Smith & S. Harland

*Lung Unit, Department of Medicine, Royal Marsden Hospital, Downs Road, Sutton, Surrey, SM2 5PT, UK.*

Carboplatin (JM8) is a new *cis*-platin analogue with a similar spectrum of clinical activity to the parent compound but without nephrotoxicity or neurotoxicity, and with better patient tolerance. In this phase II study the activity of JM8 was assessed against mesothelioma (M), for which conventional treatment is usually ineffective. Seventeen patients (pts) with symptomatic M (13 pleural, 4 peritoneal) were treated with JM8 300–400 mg m$^{-2}$ i.v. q. 28 days. Fourteen were males and 3 females, median age was 57 (range 22–78 yrs). All pts had evaluable disease on chest X-ray, CT scan or had measurable subcutaneous nodules, and were staged according to the modified schema of Butchart (Antman, K.H. *Sem. Oncol.*, **8**, 313, 1981). Three patients had stage (S)

I disease, 8 pts SII, 4 pts SIII and 2 pts SIV. Prior therapy included chemotherapy in 3 pts and radiotherapy in 4 pts. One patient with peritoneal SI achieved a CR (15 months duration) and 1 pt with pleural SII achieved a PR (11 months duration), (overall response rate 2/17; 12%; 95% confidence limits 0–27%). Four other pts with pleural M (24%) had marked symptomatic relief of pain or dyspnoea lasting 1, 3+, 4 and 14 months. All pts who achieved either an objective or a subjective response were previously untreated. All responses or relief of symptoms began to occur within 2 courses of treatment. In general therapy was well tolerated. Toxicity (WHO Grade) was as follows: Nausea/vomiting grade I 5 pts, II 7 pts, III 1 pt; leucopenia I–II 0 pts, III 1 pt; thrombocytopenia I 1 pt; anaemia II 1 pt. JM8, like other cytotoxic drugs, is active only in a small minority of pts with mesothelioma but its low toxicity and ability to achieve symptomatic relief may justify a therapeutic trial of up to 2 courses, with further treatment reserved for patients achieving clinical benefit.

### Phase II studies of a novel antifolate CB3717, and the platinum analogues JM8 and JM9, in mesothelioma of pleura and peritoneum

B.M.J. Cantwell, A.L. Harris & S. Ghani

*University Department of Clinical Oncology, Regional Radiotherapy Centre, Newcastle General Hospital, Newcastle upon Tyne, UK.*

The objective response rate of malignant mesothelioma to cytotoxic chemotherapy is low and 'standard' chemotherapy does not exist. We treated 18 patients with inoperable progressive mesotheliomas (17 pleural, 1 peritoneal) with CB3717 (an inhibitor of thymidylate synthetase) i.v. 3 weekly as first systemic treatment. One patient had an objective partial response. CB3717 toxicities included reversible hepatotoxicity (transaminitis), nausea, lassitude, skin rashes, conjunctivitis and one hypersensitivity-type reaction.

Thirteen patients were given platinum analogues, 8 receiving JM8 and 5 JM9 as part of a multinational phase II comparative study. Two of 8 JM8 treated patients had prior CB3717, 3 of 5 JM9 treated patients had prior CB3737. In 8 JM8 treated patients 2 objective partial responses occurred (one patient previously responded to CB3717). Toxicities of JM8 and JM9 were similar: emesis in first 24 h, myelosuppression and diarrhoea in some JM9 treated patients. Both drugs were easily administered by i.v. infusion once monthly on an out-patient basis. JM8 emerges as active in mesothelioma and study continues. Primary drug resistance is a major obstacle to successful treatment of mesothelioma and phase II studies of novel agents should be first line treatment.

Drug doses in these studies were: CB3717 300–400 $mg\,m^{-2}$; JM8 400 $mg\,m^{-2}$; JM9 300 $mg\,m^{-2}$, with dose modifications for toxicity. Patients received a maximum of 6 courses of any drug.

### High-dose ifosfamide in small cell lung cancer

H.M. Warenius[1], D.C. Hurman[2] & B. Cottier[2]

[1]*Liverpool University Department of Radiation Oncology, Clatterbridge Hospital and* [2]*Mersey Regional Centre for Radiotherapy and Oncology, Clatterbridge Hospital, Bebington, Wirral, L63 4JY, UK.*

Nineteen previously untreated patients with either limited-disease (LD-SCLC) or extensive-disease small cell lung cancer (ED-SCLC) received single-agent high dose (HD) ifosfamide (8 $gm\,m^{-2}$) as a 24 h infusion with concurrent administration of mesna. This treatment cycle was repeated at an interval of 28 days.

Haematological toxicity was minimal with neutropenic septicaemia occurring in only 1 patient. Mesna was effective in preventing urothelial toxicity.

Following 2 cycles of HD-ifosfamide, in 17 radiologically evaluable patients, there were 2 complete responses (CR) and 11 partial responses (PR) giving an overall response rate (CR+PR) of 76.5%. Previous series have reported only a 50–60% overall response rate (Souhami, *Practitioner*, **227**, 1553, 1983; Aisner *Proc 13th Int Cong. Chemother. Vienna*, **228**, 19, 1983) to conventional-dose ifosfamide.

Our results represent a significant improvement in response rate, indicating a possible dose-response relationship for ifosfamide in the treatment of SCLC.

### Serum CA 125 and CA 153 as tumour markers in clinical practice

K. Patel[1], D. Parker[1], P. Harnden-Mayor[2] & B. Naylor[2]

[1]*Department of Medical Oncology and* [2]*Department of Pathology, Bradford Royal Infirmary, Bradford BD9 6RJ, UK.*

Our experience of CA 125 as an ovarian tumour marker at Bradford Royal Infirmary is presented.

A total of 56 patients including 36 with ovarian cancer were followed up. Six of 7 (85%) patients with measurable tumour bulk had a positive correlation between serum CA 125 level and tumour response. In 6/20 (30%) of patients with non-ovarian malignancy elevated levels of CA 125 were found. Concurrent assay of CA 153 discriminated between breast and ovarian carcinoma in 27/28 patients. CA 125 appears a reliable marker for ovarian carcinoma. The ratio between CA 125/CA 153 levels allows discrimination between breast and ovarian carcinoma. The minimum detectable tumour bulk with these markers remains to be established.

**The radiological contribution to the staging of anaplastic testicular germ cell tumours (AGCT): Does lymphography have a role?**

S.M. Crawford[1], B.K. Wignall[2], J.E. Boultbee[2], J. McIvor[2], J.A. Ledermann[1], E.S. Newlands[1] & R.H.J. Begent[1]

*Departments of [1]Medical Oncology and [2]Diagnostic Radiology[2], Charing Cross Hospital, London W6 8RF, UK.*

Accurate staging is essential in the management of AGCT, especially when a surveillance-only policy for Stage I tumours is pursued. Traditionally this has included computed tomography (CT) and lymphography (LG). LG is a time-consuming and uncomfortable procedure. We have assessed its diagnostic yield in comparison with that of CT in respect of para-aortic lymphadenopathy (PAL).

The radiological records were reviewed of 38 patients in whom CT had been performed at Charing Cross Hospital and who had LG either performed or reviewed here. In 2 of 5 patients with a positive CT, the LG was negative. Of 33 in whom CT was negative, the LG confirmed PAL in only 1. When tumour markers are taken into account, no patient's management was altered by LG.

In patients assigned to Stage I follow-up, the relapse rate among those who had had LG was 29% compared with 9% among those who had not ($\chi^2 = 2.64$, NS).

Ultrasound is highly concordant with CT in assessing PAL and is much more sensitive in detecting liver metastases. We suggest that it, rather than LG, is the investigation of choice to complement CT.

**Gastric cancer: Prognostic indicator of survival**

D. Cunningham[1], D. Hole[3], D.J. Taggart[2], D.C. Carter[2], M. Soukop[1] & C.S. McArdle[2]

*[1]Department of Medical Oncology and [2]University Department of Surgery, Glasgow Royal Infirmary and [3]the Cancer Surveillance Unit, Ruchill Hospital, Glasgow, UK.*

We have reviewed the records of 328 cases of gastric cancer treated in the Royal Infirmary, Glasgow between January 1974 and December 1984. Of these patients, 128 (39%) had a curative resection (CR), 32 (9.8%) had a palliative resection (PR), 33 (10.0%) had a gastroenterostomy (G), 26 (7.9%) had a celestin tube inserted (CT), 58 (17.7%) had a laparotomy alone (LA) and 51 (15.5%) had no surgical procedure (NS). Operative mortality for the CR group has dropped from 17.6% between 1974–79 to 8.4% between 1980–84.

The 5 year survival (YSu) for all patients was 11%, but all long term survivors (except 1 patient) came from the CR group who had a 5YSu of 24%. The best predictor of long term survival for the CR group was serosal involvement ($P < 0.001$).

Also, patients with a long duration of symptoms (>6 months) survived longer (5YSu = 51%) than those with a short history (<6 months; 5YSu = 14%) $P = 0.001$. Of the patients who did not have a curative procedure: the median survival for the PR group was 6 months which was significantly better ($P < 0.05$) than the survival of the groups; G 4 months, CT 2 months, LA 2 months and NS 1 month.

This analysis defines the prognostic indicators for survival of patients with gastric cancer. A subgroup of patients with a long duration of presenting symptoms, who ultimately have a good prognosis, has been identified and these findings may reflect an intrinsic difference in 'tumour biology'.

**Six drug alternating combination chemotherapy in the treatment of poor prognosis non-Hodgkin's lymphoma**

J.A. Green

*CRC Department of Radiation Oncology, Clatterbridge Hospital, Wirral, Merseyside, L63 4JY, UK.*

There have been several reports of attempts to improve the response rate and survival in non-Hodgkin's lymphomas of aggressive histological type by dose escalation, increase in number of drugs or schedule manipulation. Etoposide has recently been shown to be active in this tumour and had been incorporated in an intensive 6 drug regime. Twenty-two patients were treated with adriamycin 50 mg m$^{-2}$ i.v. vincristine 1.4 mg m$^{-2}$ day 1, methotrexate 300 mg m$^{-2}$ days 7, 14, 28, 35, cyclophosphamide 600 mg m$^{-2}$ i.v. day 21, etoposide 120 mg m$^{-2}$ i.v. days 21–23, and prednisolone 60 mg m$^{-2}$ oral days 1–5 and 21–25. The mean age was 48 (range 29–71), and the stages were I: 2, II: 13, III: 3, IV: 4. Two patients had received prior radiotherapy and none previous chemotherapy. Consolidation radiotherapy was given to sites of bulk disease in 3 patients. A total of 4 course (24 weeks) was planned and 15 patients received this number. Toxicity was acceptable with one patient transferred to an alternative regime after 1 cycle, 6 episodes of neutropenic fever and no toxic deaths. Of 19 evaluable patients, there have been 15 complete remissions and 4 partial remissions. Three patients have died, at 3 months (mesenteric artery thrombosis), and 2 at 9 months (relapsed disease resistant to second-line treatment). Median follow-up is 1 year with 5 patients alive and well at over 2 years. The high response rate of this combination with acceptable toxicity is encouraging.

**Methyl prednisolone, VP16, vindesine and chlorambucil (PEEC) as initial or salvage therapy for non-Hodgkin's lymphoma (NHL)**

R.C.F. Leonard and S.J. Proctor on behalf of the East of Scotland and Newcastle Lymphoma (ESNLG) Working Party on Therapy (J.S. Scott, N.C. Allan, A. Parker, A. Dawson, G. Ritchie, T. Sarkar, R. Prescott, H. Lucraft & A. Harris)

We have recently completed a pilot study of a novel combination of drugs, PEEC, (P 500 mg m$^{-2}$; VP16 100 mg m$^{-2}$ i.v.; vindesine 3 mg m$^{-2}$ i.v.; chlorambucil 30 mg m$^{-2}$ p.o. day 1; VP16 200 mg m$^{-2}$; chlorambucil 30 mg m$^{-2}$ days 2 and 3 repeat xq 3/52) with the aim of identifying an active and acceptably toxic regime with potential as salvage therapy or for alternating with CHOP-like combinations in the initial treatment of advanced high grade (HG) NHL. Thirty-two patients (pts), 19 at Newcastle and 13 at Edinburgh, were given PEEC or PEEC-CHOP as initial therapy (IT, 23 pts) or salvage (S, 9 pts). Twenty-six of 32 had HG pathology and 1 stage IE (B) 5 stage II, 10 stage III and 16 stage IV; 18 had B symptoms. All pts are included in the analysis of activity and toxicity. For PEEC alone (11 pts) the objective response (OR) was 64% (27% CR) whereas for PEEC-CHOP the OR was 71% (52% CR). Analysis of IT pts alone shows 6/7 OR for PEEC and 15/16 OR (69% CR) for PEEC/CHOP. Toxicity for PEEC was mainly alopecia 9 WHO grade III) with minor myelotoxicity and grade I/II nausea and vomiting. Toxicity for PEEC/CHOP was conveyed mainly by CHOP. Follow-up is early for survival analysis. The promising activity of PEEC/CHOP for HGNHL has initiated a multi-centre (ESNLG) phase III trial comparing the strategy of alternating the combination with bleomycin (B) and methotrexate (M) (i.e. CHOP-M/PEEC-M) with B CHOP M alone and early salvage PEEC-M.

**Evidence of chronic bone marrow damage in patients treated with MVPP for Hodgkin's disease**

J.A. Radford[1], N.G. Testa[2] T.M. Dexter[2] & D. Crowther[1]

*Departments of [1]Medical Oncology & [2]Experimental Haematology, Christie Hospital & Paterson Laboratories, Wilmslow Road, Manchester, UK.*

Acute myelosuppression is a common cause of treatment delay or curtailment in patients undergoing chemotherapy for malignant disease. Bone marrow (BM) regeneration quickly restores a picture of apparent normality once treatment is complete but evidence of persisting BM damage can be found co-existing with a normal blood picture, both in experimental systems and in patients (Testa *et al., Anticancer Res.*, **5**, 101, 1985). The BM of patients in complete remission from Hodgkin's disease (HD) with a normal peripheral blood count and at least one year after the completion of MVPP therapy have been compared with two groups of controls (BM from surgically excised ribs and from patients with HD at diagnosis) using the technique of long term bone marrow culture *in vitro*. Differences have been observed between the treated group and controls in both the morphology of the stromal cell adherent layer and in the production of haemopoietic progenitor cells (granulocyte macrophage colony forming cells, GM-CFC) with a marked reduction in the MVPP treated group. These results suggest chronic BM damage in the treated individuals, and experiments are in progress to determine the relative involvement of stem cells and the supporting stroma.

### New bone formation seen during chemotherapy

G.J. Forrest[1], N.L. Gilchrist[1], B. Boyce[2], D. Cunningham[1], I.T. Boyle[3] & M. Soukop[1]

[1]*Department of Medical Oncology,* [2]*Department of Pathology and* [3]*Department of Pathology, Royal Infirmary, Glasgow G4 0SF, UK.*

Combination chemotherapy is known to affect the growth rate of several human tissues. Six patients undergoing chemotherapy for Hodgkin's Disease (HD) were investigated to see whether the administration of combined chemotherapy affected the rate of new bone formation. Patients received a bone biopsy prior to the start of therapy and after six pulses of chemotherapy with mustine 6 mg m$^{-2}$, vincristine 1.4 mg m$^{-2}$, hydrocortisone 100 mg, all given on days 1 and 8 with procarbazine 100 mg and prednisolone 40 mg given daily by mouth days 1–14, cycled every 6 weeks. Only one patient showed bone involvement with HD prior to therapy. Four patients showed marked osteopaenia with decreased bone trabeculae prior to therapy. After six pulses of chemotherapy it was found the four patients with marked osteopaenia previously, had shown marked new bone formation during the period of treatment. No change in bone formation was found in the other two patients. No change in any biochemical marker could be found between pre and post treatment values. These results are contrary to what might have been expected and the cause of this phenomenon is not known, but may be due to local hormonal activity within bone.

### A new marker of disease for malignant lymphoma

B.W. Hancock[1], L. Bruce[1], L. Cawood[1] & S. Metcalfe[2]

[1]*Department of Medicine, Royal Hallamshire Hospital, Sheffield and* [2]*Department of Surgery, Addenbrooke's Hospital, Cambridge, UK.*

Patients with malignant lymphoma – 144 with non-Hodgkin's lymphoma (NHL) and 132 with Hodgkin's disease (HD) – have been monitored by both erythrocyte sedimentation rate (ESR) and the B5 test, in addition to standard clinical and radiological assessments. The B5 test involves haemagglutination by a monoclonal antibody designated B5 (Metcalfe *et al., Br. J. Cancer,* **49,** 337, 1984).

Of those patients who were well and in remission, 41/155 (26%) showed some B5 positivity: this compared to an incidence of 20% in non-tumour bearing individuals (122/551). In contrast, those patients with persistent active disease, or in relapse, showed a much higher incidence of B5 positivity (84%, 99/118).

Combination of ESR and B5 gave an increased specificity for active disease in that 35/40 (87%) of patients who were B5 positive and had a raised ESR, also had active disease; marker negative patients with a normal ESR rarely showed active disease (9/180; 8%).

Serial monitoring of 113 patients showed that B5 status often followed tumour status, becoming negative in remission. Overall, B5 used in combination with ESR improved both the specificity and sensitivity of monitoring malignant lymphoma over that of either test alone. These findings suggest a role for the B5 test to be included in the clinical monitoring of HD and NHL patients.

### Intermediate lymphocytic lymphoma – A possible origin in mucosa associated lymphoid tissue

P.A. Daly, D.S. O'Briain, F. Rogers, E. Lawlor, C. Blaney & A.J. O'Brien

*Departments of Clinical Haematology-Oncology and Histopathol, St James's Hospital and Trinity College, Dublin 8, Eire*

Seven patients with malignant lymphoma lymphocytic of intermediate-cell type (ILL) were studied over 4 yrs. Three patients presented with gastrointestinal (GIT) disease and 2 with disease of the urinary bladder. One of the latter had extensive GIT disease which was asymptomatic and a further patient had radiological evidence of disease in the terminal ileum. The remaining patient had a solitary extra-nodal lesion above the rt. breast which developed during pregnancy. There were 5 males and 2 females, age range 33–60 yrs – median 46. Three patients with GIT involvement fulfilled the criteria for multiple lymphomatous polyposis (MLP) of the GIT. Other sites of involvement included abdominal and peripheral lymph nodes, bone marrow, peripheral blood, spleen, liver, lung and meninges. The histology was diffuse in 3 and follicular in 4 where the growth pattern was of a mantle zone lymphoma. An 8th pt, male, aged 71 years, with MLP had the histologic picture of poorly differentiated lymphocytic lymphoma (PDLL) diffuse, but had an immunologic phenotype which was similar to that found in patients with ILL. Immunohistochemistry on cryostat sections from 5 pts showed strong monotypic Ig staining, HLA DR+ve., B1+ve., T10−ve and variable reaction with CALLA, T1 and FMC7. Peripheral blood of 4 patients also showed a B-cell clonal expansion (B1+ve., SmIg+ve., HLA DR+ve., FMC7+ve). Involvement of mucosal surfaces, especially the GIT and urinary tract, is common in this lymphoma with a distinctive histology and immunologic phenotype suggesting a possible origin in mucosa associated lymphoid tissue (MALT). This should be taken into consideration when staging patients with this particular non-Hodgkin's lymphoma because of the obvious therapeutic implications and further studies should help to clarify the mucosal immunologic system.

### Incidence and tramission of human T-cell leukaemia lymphoma virus (HTLV-1) in the UK

A.G. Dalgleish[1], E. Matutes[2], D. Catovsky[2], R.A. Weiss[1], T.A. Lister[2], S. Pegram[4] & M.F. Greaves[4]

[1]*Institute of Cancer Research Fulham Road, London SW3 6JB,* [2]*MRC Leukaemia Unit, Royal Postgraduate Medical School, Hammersmith Hospital, London W12,* [3]*St Bartholomew's Hospital, London EC2 and* [4]*Leukaemia Research Fund (Institute of Cancer Research), UK.*

HTLV-1 is the causative agent of adult T-cell leukaemia/lymphoma (ATLL) which was first recognised in the UK as occurring in people of Caribbean origin (*Lancet,* **i**, 639, 1982), an observation which led to the recognition that the Caribbean basin, like Japan, was endemic for HTLV-1 infection.

We have screend over 500 sera from communities with large Caribbean immigrant community and found 19 seropositive asymptomatic people. Studies on the family members have revealed marked clustering within families. In one case a husband and wife and 2 eldest children (over 20 years) were seropositive whilst their youngest 2 children were seronegative. Both the seropositive children were born in the UK and one had never been abroad. Following short term lymphocyte culture, virus was detected by specific monoclonal antibodies to HTLV-1 (p19 and p24) and by electron microscopy in 4 of the healthy seropositive people. These data support that HTLV-1 has a very low incidence in the UK, outside the Caribbean Community, as well as that the most likely modes of transmission in this country is sexual, blood to blood and mother to child.

Two cases of HTLV-1 antibody positive ATL in white Caucasians in the UK with no known contacts or risk factors suggest that white Caucasians may be more susceptible to HTLV-1 infection and have a short mean incubation time to the development of ATLL compared with Japanese and Afro-Caribbean peoples. Other unreported cases support this hypothesis (R.C. Gallo, personal communication).

### Prognostic indicators in patients with high grade histology non-Hodgkin's lymphoma receiving VAP

R.A. Cowan[1], J.M. Jones[2] & D. Crowther[1]

[1]*CRC Department of Medical Oncology and* [2]*Department of Medical Statistics, Christie Hospital & Holt Radium Institute, Manchester M20 9BX, UK.*

We have studied 200 patients with high grade non-Hodgkin's lymphoma treated by the Manchester Lymphoma Group between 1975 and 1985 to ascertain which factors are useful in predicting disease outcome. The cohort comprises patients with localised and widespread disease and all have been treated in protocols incorporating weekly chemotherapy with VAP (vincristine, adriamycin, prednisolone). The median follow-up for the group is $5\frac{1}{2}$ years.

Prognostic variables have been examined using the logrank test and multivariate analyses (discriminant method and Cox's model).

Factors independently associated with an improved overall survival were: attainment of complete remission (CR), $P=<0.000001$, a serum LDH within the normal range, $P=0.0005$, clinical stage I disease, $P=0.018$ and centroblastic histology (Liel classification), $P=0.034$. Excluding remission status from the analysis, clinical stage became the most important indicator of prognosis, $P=<0.0001$, followed by serum albumin, patient age and Gamma GT. Having shown attainment of CR to be so important in determining survival, discriminant analysis was performed to ascertain which factors predicted the likelihood of achieving CR: clinical stage and serum albumin were equally important followed by B symptoms and bulk disease. Multivariate analysis was also carried out to assess which factors were predictive of survival in patients who had achieved CR and this revealed clinical stage, Gamma GT and centroblastic histology to be significant.

These results concur with other smaller published series and it is hoped that such prognostic information will prove useful in selecting which patients should be treated with the more intensive chemotherapy currently advocated for high grade non-Hodgkin's lymphoma.

**N-acetyl-b-d-glucosaminidase (B NAG) as an early marker of *cis*-platin-induced renal tubular dysfunction**

M. Teeling[1], B. Casey[2], I. Pratt[2], M. Ryan[2] & D.N. Carney[1]

[1]*Medical Oncology Unit, Mater Hospital, Dublin 7 and* [2]*Department of Pharmacology, UCD, Eire*

The long term use of the cytotoxic agent *cis*-platin (P) is limited by its nephrotoxicity. To assess its value as an early marker of renal tubular dysfunction sequential urinary levels of the lysosomal enzyme B NAG were measured in 20 patients receiving P chemotherapy (CT) for a variety of malignancies. Levels of B NAG were measured in pre and post treatment urine samples and compared with urinary excretion of magnesium (Mg), plasma Mg and standard parameters of renal function. Following P treatment, a marked elevation in B NAG excretion was seen in all patients, reaching a peak level between 1–5 days post treatment, and returning to base line levels by 7–9 days. Greater levels of B NAG excretion were seen in the first course of CT than in subsequent courses, while plasma creatinine and urea values remained within the normal range. Urinary excretion of Mg was within the normal range during the first course of CT but Mg wasting was frequently seen in later courses of CT, associated with hypomagnesaemia. Persistently elevated levels of B NAG were observed in one patient who later developed renal insufficiency. Peak excretions of B NAG were dissociated in time from maximum Mg excretion. These results suggest; (1) Urinary excretion of B NAG is a sensitive indicator of P nephrotoxicity, (2) Persistently elevated urinary excretion of B NAG predicts for early renal dysfunction and (3) the dissociation in time of Mg wasting from B NAG excretion may not be directly related to the P-induced proximal tubular damage.